# What drives adoption of a computerised, multifaceted quality improvement intervention for cardiovascular disease management in primary healthcare settings? A mixed methods analysis using normalisation process theory

**DOI:** 10.1186/s13012-018-0830-x

**Published:** 2018-11-12

**Authors:** Bindu Patel, Tim Usherwood, Mark Harris, Anushka Patel, Kathryn Panaretto, Nicholas Zwar, David Peiris

**Affiliations:** 1 The George Institute for Global Health, University of New South Wales, Sydney, New South Wales Australia; 2 University of Sydney, Sydney, New South Wales Australia; 3 University of New South Wales, Sydney, New South Wales Australia; 4 University of Queensland, Brisbane, Queensland Australia; 5 University of Wollongong, Wollongong, New South Wales Australia

**Keywords:** Quality improvement, Health information technology, Primary healthcare, Health service, Normalisation process theory, Process evaluation, Mixed methods, Adoption

## Abstract

**Background:**

A computerised, multifaceted quality improvement (QI) intervention for cardiovascular disease (CVD) management in Australian primary healthcare was evaluated in a cluster randomised controlled trial. The intervention was associated with improved CVD risk factor screening but there was no improvement in prescribing rates of guideline-recommended medicines. The aim of this study was to conduct a process evaluation to identify and explain the underlying mechanisms by which the intervention did and did not have an impact.

**Methods/design:**

Normalisation process theory (NPT) was used to understand factors that supported or constrained normalisation of the intervention into routine practice. A case study design was used in which six of the 30 participating intervention sites were purposively sampled to obtain a mix of size, governance, structure and performance. Multiple data sources were drawn on including trial outcome data, surveys of job satisfaction and team climate (68 staff) and in-depth interviews (19 staff). Data were primarily analysed within cases and compared with quantitative findings in other trial intervention and usual care sites.

**Results:**

We found a complex interaction between implementation processes and several contextual factors affecting uptake of the intervention. There was no clear association between team climate, job satisfaction and intervention outcomes. There were four spheres of influence that appeared to enhance or detract from normalisation of the intervention: organisational mission and history (e.g. strategic investment to promote a QI culture enhanced cognitive participation), leadership (e.g. ability to energise or demotivate others influenced coherence), team environment (e.g. synergistic activities of team members with different skill sets influenced collective action) and technical integrity of the intervention (e.g. tools that slowed computer systems limited reflective action).

**Discussion:**

Use of NPT helped explain how certain contextual factors influence the work that is done by individuals and teams when implementing a novel intervention. Although these factors do not necessarily distil into a recipe for successful uptake, they may assist system planners, intervention developers, and health professionals to better understand the trajectory that primary health care services may take when developing and engaging with QI interventions.

**Trial registration:**

ACTRN 12611000478910. Registered 08 May 2011.

**Electronic supplementary material:**

The online version of this article (10.1186/s13012-018-0830-x) contains supplementary material, which is available to authorized users.

## Background

In the area of cardiovascular disease (CVD) risk management, around 50% of adults attending primary healthcare are adequately screened for CVD risk and only around 40% of those identified at high risk are prescribed recommended medications [1–3]. To address these entrenched gaps, the US National Academy of Medicine recommended changing the healthcare environment in four ways: increasing the uptake of evidence in healthcare delivery, leveraging information technology, aligning payment reform with quality improvement and enhancing workforce support [4, 5].

Quality improvement (QI) is a well-established process to improve the efficiency and processes of healthcare with goals of achieving sustained improvements in health outcomes and system performance [6, 7]. Three inter-related QI strategies are pertinent to this paper. The first is the Chronic Care Model, particularly the sub-domains of decision support and optimising clinical information systems. Several evaluations of varying degrees of quality have demonstrated improvements in processes of care and patient health outcomes with this model [8, 9]. The second is the Breakthrough Series Collaborative model organised around principles of closing evidence-practice gaps, minimising unwanted variation in care, disseminating and diffusing best practice activities, fostering collaborative work ethics, and implementing rapid evaluation and action (plan-do-study-act) cycles [10, 11]. There have been relatively few randomised evaluations of collaborative models, and outcomes have been mixed [12]. Third, health information technology (HIT) is a key enabler of the Chronic Care Model and the collaborative model to improving QI. HIT strategies with the strongest evidence base include computerised clinical decision support systems and audit and feedback of performance to providers. These have been shown to improve processes of care with a modest impact on healthcare outcomes [13–17].

### Development of the intervention

Drawing on the above literature, we developed a multifaceted QI intervention for CVD management in Australian primary healthcare. Additional file 1 details the development and validation of the intervention, and additional details can be found elsewhere [18]. The intervention, named ‘HealthTracker,’ included real-time decision support integrated with electronic medical records; an interactive CVD risk communication tool between provider and patient; an automated clinical audit tool which provided performance feedback; and a web portal which provided peer-ranked performance trends.

### Clinical effectiveness evaluation

The intervention was evaluated between September 2011 and June 2013 in the TORPEDO (Treatment of Cardiovascular Risk in Primary Care Using Electronic Decision Support) study—a cluster-randomised controlled trial (cRCT) involving 38,725 people at 60 sites (40 general practices and 20 Aboriginal Community Controlled Health Services (ACCHSs)) [18]. At completion of the cRCT, the intervention was provided for a further 18 months to the end of 2015 to both intervention and control sites. The primary outcomes of the cRCT and the post-trial period have been previously published and related to guideline-recommended CVD risk factor screening and prescribing of recommended medicine to those identified at high CVD risk [19, 20]. Table 1 summarises the findings. The key findings were that the intervention was associated with improved CVD risk factor screening but there was no improvement in prescribing rates of guideline-recommended medicines.

**Table 1:** Summary of the TORPEDO trial and post-trial results

TORPEDO trial (17.5 months follow-up)	• 25% relative improvement in CVD risk factor screening • No significant difference in prescribing rates of recommended medicines for people at high CVD risk • High CVD risk individuals not prescribed optimal recommended treatment at baseline, intervention was associated with 33% relative improvement in prescribing rates
Post-trial phase (18 months follow-up)	• Plateauing of improvement in screening of CVD risk factors • Ongoing improvement in prescribing of recommended medicines in both the intervention and usual care arm

### Support and training

The intervention services (referred henceforth as ‘sites’) received introductory training visits from staff in the use of the software via face to face visits and webinars. A technical help desk was available for software-related problems. During the post-trial period, this support was scaled back and mainly restricted to software installation [20]. Software licences and technical support were provided free to intervention sites during the trial period and to all sites participating in the post-trial phase. Patient and practice costs associated with patient care occurred as per usual practice.

The objective of this study was to conduct a process evaluation of the TORPEDO trial to identify the underlying mechanisms by which the intervention did and did not have an impact on trial outcomes amongst sites participating in the study. It forms part of a broader multimethod process evaluation in which several studies are being conducted to examine the implementation and impact of the intervention.

## Methods and design

The process evaluation was designed prior to the commencement of the cRCT by a project working group which comprised researchers involved in the trial development and external researchers not involved in the intervention. The logic model, which articulates all the component studies of the process evaluation, has been previously published and is included here as Additional file 2 [21]. This paper refers to study 2 in that model.

### Sample and setting

We used a case study design to explore implementation processes at each site. This approach enabled us to answer ‘how’ and ‘why’ questions drawing on multiple data sources [22, 23]. ‘Cases’ refer to trial intervention arm sites who agreed to participate in this study. In determining case selection, we purposively invited eight intervention sites toward the end of the trial phase that exhibited a broad variation in trial primary outcomes, the number of staff at each site and type of site (general practice versus ACCHS, urban versus rural and size). Six intervention sites agreed to participate (four general practices and two ACCHSs). We also compared quantitative data in the cases with ‘non-case intervention sites’ (intervention arm sites not selected as cases) and ‘control sites’ (sites assigned to the usual care arm of the cRCT).

### Theoretical framework

We used a framework (the UK Medical Research Council (MRC)) guidance on process evaluations for complex interventions [24] and a theory (normalisation process theory (NPT)) [25] to understand the mechanisms involved in implementation of the intervention. The MRC framework provides practical guidance on designing and conducting evaluations to assess implementation (fidelity, dose and reach) of complex interventions, explain causal mechanisms (how change is produced) and identify contextual factors (anything external to the intervention) associated with variation in outcomes [26, 27]. NPT seeks to understand the implementation processes and the extent to which an intervention becomes ‘normalised’ in the service environment [25, 28]. NPT is focused on the work people do individually and collectively to implement, embed and integrate new interventions into their physical and social context. This is characterised by four generative mechanisms of coherence (‘what is the work?’), cognitive participation (‘who does the work?’), collective action (‘how does the work get done?’) and reflexive monitoring (‘how is the work understood?’). By embedding NPT within the MRC framework, we sought to understand the interaction between health service context, the generative mechanisms related to the implementation of the intervention and the outcomes observed in the cRCT.

### Data sources and collection

Table 2 outlines the quantitative data sources.

**Table 2:** Quantitative data sources

1. To assess effectiveness of the intervention on the trial outcomes within sites, data from electronic medical records were collected using a validated extraction tool at baseline, end of trial and end of post-trial phase as part of the TORPEDO trial. 2. To assess the support requirements provided by the project staff, support time was calculated based on contact time logged by both the technical helpdesk and the research team. Support time varied depending on availability and number of staff, staff requests and technology-related troubleshooting. 3. To assess acceptability and fidelity of the intervention, staff were invited to complete three surveys toward the end of cRCT: i. An end of study mail survey for general practitioners who were part of the intervention sites was developed by the research team to assess acceptability and fidelity of the intervention. The questions were focused on satisfaction with the intervention components, recommendations of evidence-based guidelines, the intervention’s effect on the quality of care, and frequency of use. In addition, there were questions about the practice characteristics and personal use of information technology. It was reviewed for content validity by the PWG. Although we had intended to look at usage analytics to look at intervention fidelity, due to technical problems with the software database, we were unable to generate accurate usage logs and therefore had to rely on staff self-report. Nine GPs within the six cases completed the survey, and 23 GPs from 15 non-case intervention sites completed the survey. The findings from this survey have been published and used in this paper as complementary data [20]. ii. Drawing on the NPT sub-domain of ‘collective action’ in which team members work together to incorporate innovation into practice, a team climate inventory (TCI)* survey was administered. This is a 44-item questionnaire which assesses team vision (11 items), participative safety (12 items), task orientation (7 items), support for innovation (8 items) and social desirability (6 items) with each item rated on a 5-point Likert scale. iii. In order to assess if job satisfaction may be an influential factor in driving outcomes, the Warr-Cook Wall Job Satisfaction survey* was administered. Based on previous work, this 10-item questionnaire assesses physical work conditions, income, amount of responsibility given, freedom in the job, variety, work colleagues, opportunity to use abilities, recognition and hours of work. It was adapted for use with general practices and ACCHSs using a 7-point Likert scale. * The TCI and job satisfaction surveys were either distributed together by mail or in person during the end of trial data collection period. Sites were followed up 1 week later by telephone on expected completion timeframe. For surveys not received within the month, a second attempt to follow-up was made. The TCI and job satisfaction surveys were completed by 68 health professionals from the six cases, 113 health professionals from 18 non-case intervention sites and 65 health professionals from 15 control sites.	

In addition to the quantitative data, semi-structured interviews were conducted by two researchers with staff between May 2013 and February 2014 at each of the cases. A diverse mix of general practitioners (GPs), nurses, managers, Aboriginal health workers (AHWs) and administrative assistants was sought. Interviews took place at the sites toward the end of the trial and during the post-trial phase to not overly influence the implementation phase of the intervention. Interview questions were aligned with NPT domains. Broad domains of inquiry included the following: (1) why health staff did/did not use the intervention, (2) how was the intervention used in routine practice, (3) how did the intervention help use of guidelines, (4) how was the intervention integrated at the site and (5) what impact did the intervention have on the way personnel do their work (Additional file 3). During the process of conducting the interviews, questions were iteratively modified to allow exploration of emergent themes identified by the project working group.

### Data analysis

We conducted a mixed methods analysis adopting an explanatory sequential design whereby quantitative data were initially tabulated and qualitative data were analysed to gain better understanding of the processes of implementation and the resultant quantitative outcomes [29]. The systematic integration of qualitative and quantitative data within a single case allowed for detailed examination of empirical data from varied perspectives.

Contact time with sites was tabulated, and simple frequency analyses conducted. The satisfaction survey results were reported as frequencies or proportions [20]. For the team climate inventory (TCI), mean scores were calculated for each sub-domain and a total mean score was calculated across all domains (maximum score 44) [30]. For the job satisfaction survey, each of the seven domains were equally weighted and a total mean score (maximum score 7) was calculated [31, 32]. Scores were reported for each case and overall mean scores were calculated to compare the three groups for the TCI and job satisfaction surveys: case sites, non-case intervention sites and control sites. Associations between the TCI and job satisfaction survey scores and trial outcomes overall and for these three groups were analysed using univariate analyses of variance.

Interview data were organised in three stages and assisted by Nvivo 11 (QSR International Melb. Vic), a data management tool. An initial familiarisation stage was conducted in which five interviews from three cases (cases 1, 2 and 6) were analysed and discussed with the project working group. Following this, an initial thematic coding framework was developed that consisted of both descriptive codes derived from the initial thematic framework and new codes that were inductively developed as we became more familiar with the data. Thematic saturation was achieved after interviewing 19 health professionals with no new codes being created. Several meetings with the project working group were held to discuss the findings and their significance. The NPT constructs were continuously drawn on to assist with the interpretation of the findings. In particular May and Finch’s outline of the mechanisms, components and investments to understand and identify “the trajectory and outcomes of implementation process” [28, 33]; and Mair and colleagues’ meta-review of implementation of e-health interventions using an NPT-based explanatory framework was used to evaluate barriers and facilitators [34]. Key elements of the framework are summarised in Table 3.

**Table 3:**
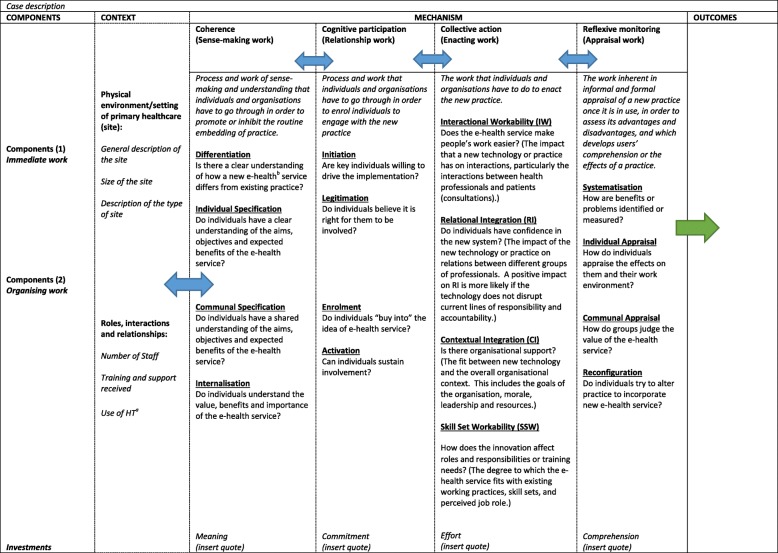
Coding framework: context and mechanism influencing outcomes of implementation of a computerised QI intervention

^a^*HT* health tracker

^b^*e-health* Electronic health

Sources:

May, C. and T. Finch (2009) [28]

Mair, F. S., C. May et al. (2012) [34]

## Results

**Fig. 1:**
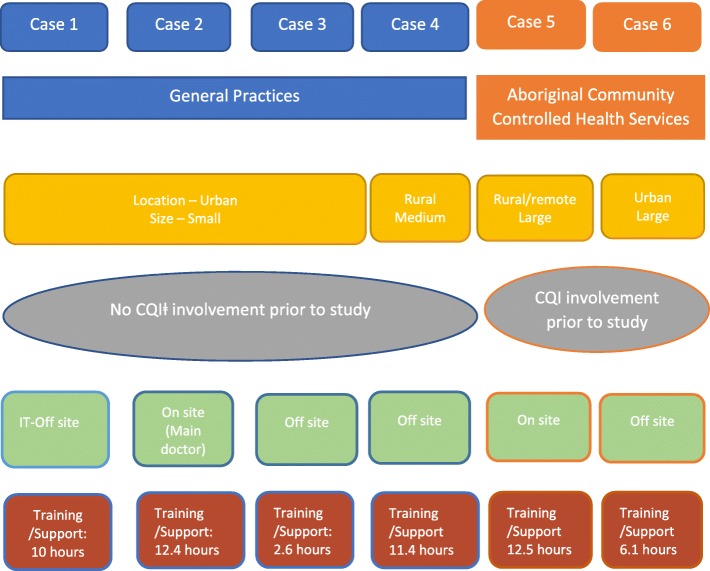
Primary healthcare service characteristics. Training/support is dependent on size of primary healthcare service, staff availability and technical issues. CQI = continuous quality improvement

**Fig. 2:**
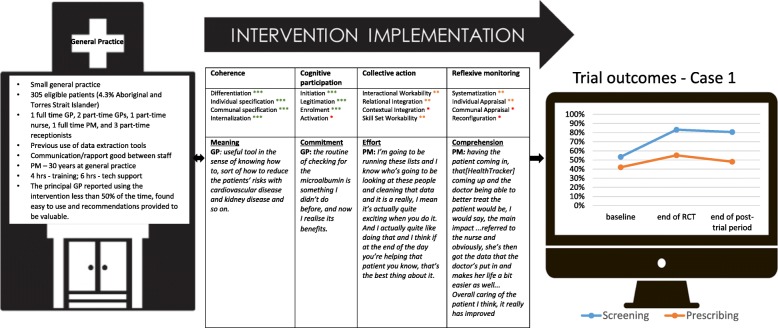
Case 1. Intervention implementation = trial period. Ratings based on health professional interview data analyses. ***Strongly present. **Partially present. *Not present

**Fig. 3:**
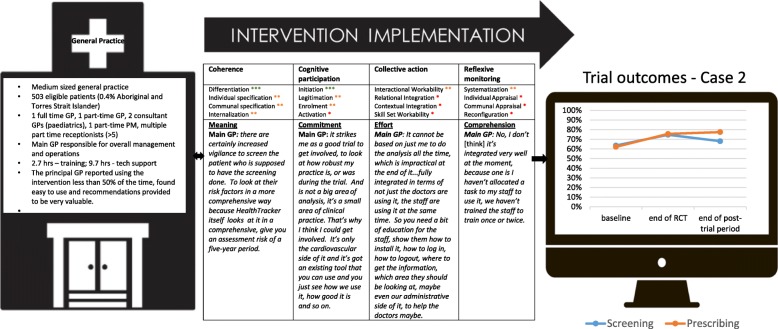
Case 2

**Fig. 4:**
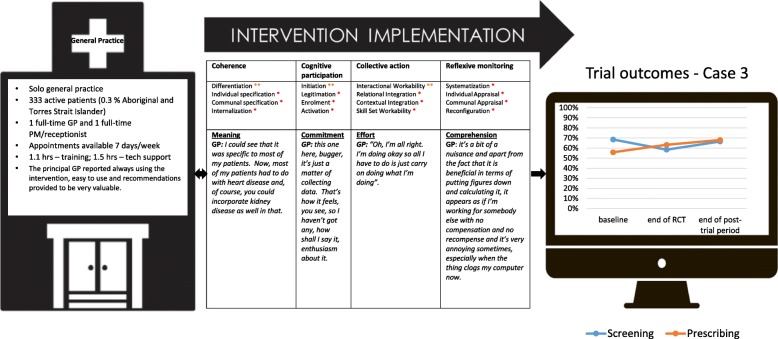
Case 3

**Fig. 5:**
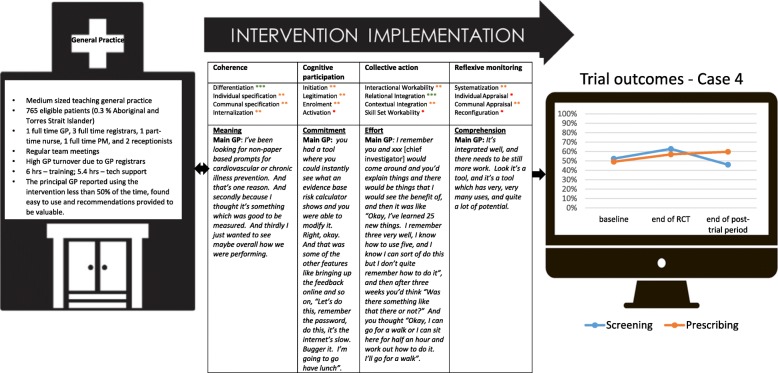
Case 4

**Fig. 6:**
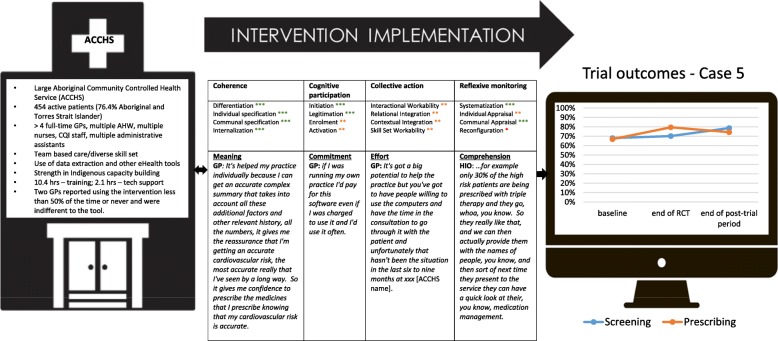
Case 5

**Fig. 7:**
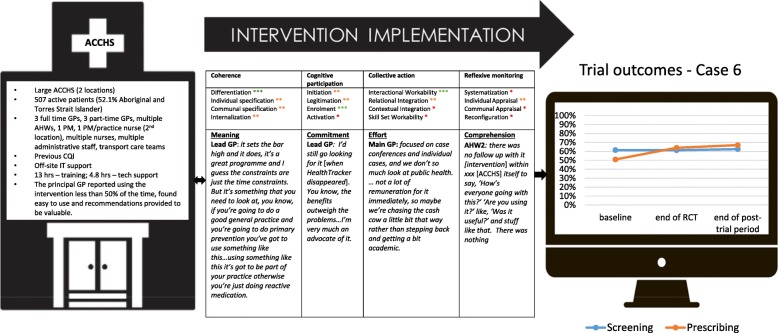
Case 6

Figure 1 summarises key characteristics of the cases, and Figs. 2, 3, 4, 5, 6, and 7 provide detailed summaries of case context, attitudes to and use of the intervention and the trial and post-trial outcomes.

Table 4 shows the mean TCI and Warr-Cook Wall job satisfaction scores for the cases, the other intervention sites, and the control arm sites. There were no statistically significant differences overall in mean TCI and job satisfaction scores between the cases and other participating intervention and usual care sites. There were also no statistically significant associations found between trial outcome variables and either the TCI or job satisfaction scores, and no heterogeneity between groups (cases, non-case intervention and control arm sites). There were also no significant associations in terms of size of sites, type of sites (general practice vs ACCHS), location and previous participation in a continuous quality improvement (CQI) program.

**Table 4:** Team climate inventory (TCI) and Warr-Cook Wall job satisfaction scores

		TCI sub-domains					Mean total TCI score (max = 44)	Mean job satisfaction score (max = 7)
	Sum of health professionals completing the surveys	Participant safety (max = 12)	Support for innovation (max = 8)	Vision (max = 11)	Task orientation (max = 7)	Social desirability (max = 6)		
Mean scores by case								
Case 1	4	9.2	6	9.8	6.5	4.5	36.0	5.7
Case 2	3	10	6.4	10	6.5	4.8	37.7	5.9
Case 3	2	9.8	6.8	9.5	6.8	5.2	38.1	6.8
Case 4	8	9.3	6.6	9.2	6.4	4.8	36.3	6
Case 5	34	9.1	6	8.4	5.8	4.6	33.9	5.7
Case 6	17	8.5	5.9	8.3	5.4	4.5	32.6	5.5
Mean score by group (cases, other intervention sites, and control sites)								
All cases (n* = 6)	68	9.3	6.3	9.2	6.2	4.7	35.8	6.0
Other intervention sites (*n* = 18)	113	8.6	5.9	8.3	5.7	4.4	33.2	5.4
Control sites (*n* = 15)	65	9.5	6.4	8.8	5.7	4.7	35.0	5.7

n* = sites

*max* maximum score

TCI and job satisfaction surveys were completed from the year 2013 during the end of trial data collection through to the end of post-trial phase

Twenty-three staff from the six cases were invited to participate with 19 agreeing to be interviewed (9 general practitioners (GPs), 4 practice managers (PMs), 3 Aboriginal health workers (AHWs), 1 practice nurse (PN), 1 health information officer (HIO), and 1 administrative assistant/practice manager (AA/PM)) (Table 5). Four GPs declined due to time constraints and/or lack of interest. Key findings for each case, organised by NPT domains, are summarised below and in Figs. 2, 3, 4, 5, 6, and 7, and detailed information on the context and outcomes for each case are in Additional file 4. More detailed findings with supporting quotes are shown in Additional file 5.

**Table 5:** Interview participants’ characteristics

	Case 1		Case 2			Case 3		Case 4		Case 5			Case 6						
Participants	GP	PM	GP	GP	PM	GP	PM/receptionist	GP	PM	GP	AHW	HIO	GP	GP	GP	PM	PM/N	AHW	AHW
Employment status																			
Full-time	x	x	x			x	x	x	x	x	x	x	x	x	x	x	x	x	x
Part-time				x	x														
Age group																			
20–29												x							
30–39							x		x	x	x				x			x	
40–49	x	x	x	x	x											x			x
50–59													x	x					
60–69						x		x									x		
70+																			
Gender																			
Male	x		x			x		x		x	x		x	x	x			x	
Female		x		x	x		x		x			x				x	x		x
Years worked in primary healthcare in Australia (years)	9	30	17	8	5	40	10	34	7	1.5	1.5	5.5	26	5.5	1	6.5	46	5	5
Length of time at current health service (years)	9	30	5	1	5	8	8	7	7	1.5	1.5	5.5	18	5.5	1	6.5	16	5	5
Given access to *HealthTracker* (yes or no)	yes	no	yes	yes	no	yes	no	yes	no	yes	no	yes	yes	yes	yes	no	yes	yes	yes

*GP* general practitioners, *PM* practice managers, *AHW* Aboriginal health workers, *HIO* health information officer, *N* nurse

*HealthTracker* intervention

### Case 1 (Fig. 2)

#### Mechanisms of implementation

The high level of understanding of the objectives (coherence) and engagement (cognitive participation) for the two main staff members (GP and PM) in combination with support from the research team strongly influenced implementation of the intervention. The GP and PM valued different aspects of the intervention—the former using the tools to enhance communication with patients, while the latter assessed aggregated data for monitoring practice performance. Despite the clear appeal of different intervention components for each of these staff, overall, there was a lack of collective action at this practice. There was little evidence of modifications to prevailing policies, procedures and resourcing. The PM was influential in encouraging the GP to use the decision support tool, providing feedback on his performance; however, other practice team members were almost completely non-engaged. Consequently, there was little evidence of enhanced interactions or relations between team members. The PM had tried to engage receptionist staff in use of the tools; however, there appeared to be little interest, possibly due to a lack of coherence for these staff. Further, there was little evidence of ongoing appraisal and evaluation of the use of the interventions, setting goals and/or strategies to overcome any barriers. These factors may provide some explanation as to why the outcomes may have declined in the post-trial setting.

### Case 2 (Fig. 3)

#### Mechanisms of implementation

The intervention had a high degree of coherence for the owner GP who was strongly interested in appraising performance at his practice compared to other general practices involved. Improvement in peer-ranked performance was the major motivation to participating in the study for this GP. However, sustained engagement was a major barrier, where major software technical issues were encountered resulting in prolonged periods where the tools were inaccessible despite multiple calls to helpdesk support. This led to sporadic engagement and diminished any possibility of systematically incorporating usage into every day work. There was virtually no evidence of collective action at this practice with staff members working in isolation of one another. Although the owner GP became highly competent in using the intervention, these skills were not transferred to other staff members. One other part-time GP expressed interest in using the tools following a training visit, but in the face of the technical barriers rapidly lost interest. Consequently, the substantial trial period improvements appear to be almost entirely attributed to the activities of the owner GP. In the post-trial period, his motivation to remain engaged was diminished and this may explain the plateau in outcomes.

### Case 3 (Fig. 4)

#### Mechanisms of implementation

The intervention lacked coherence for both the GP and PM. The GP found the initial training session to be overwhelming with trying to fit in patient consultations during the training. Although he appreciated the relevance of CVD risk screening and management, he did not readily see what value this intervention provided in addition to his usual practice. He also felt that his patients had a low level of understanding of absolute CVD risk scores and that it was not appropriate to engage them in the risk communication tools (interactional workability). Consequently, over time he saw the intervention primarily as a laborious data collection exercise with little utility and no financial compensation. This lack of coherence and cognitive participation was further compounded by technical issues midway through the trial where the decision support software appeared to be slowing down performance of his computer systems and at times cause disruptions to clinical practice (contextual integration). The PM was not encouraged to be involved in use of the intervention, and consequently, there was limited collective action evident at this practice (relational integration and skill set workability). A key driver for increased engagement was related to financial incentives. The lack of a sustainable business case meant that the work of engaging in the intervention was primarily to benefit the research team and not the practice or his patients.

### Case 4 (Fig. 5)

#### Mechanisms of implementation

The owner GP found value in the intervention as a teaching tool to train GP registrars. The GP had moderate coherence because of his specific interest in the use of tools to assess CVD risk and was interested in gauging his practice performance. Although he himself had low confidence in using computer tools in his practice, he saw it as an inevitable aspect of future clinical practice. Consequently, cognitive participation was high as a clear expectation was set by the owner GP that staff learn how to use the tools. Weekly staff meetings were used as a teaching platform and team-building forum. At these meetings, the intervention and performance outcomes were discussed intermittently throughout the trial period. The major barrier to integration across team members was the high turnover of GP registrars. The owner GP lacked capacity and confidence to train new staff, and consequently, this was left to the research team staff. The decline in screening performance in the post-trial period may in part relate to the high turnover as newer registrars commenced, and research team support was less intensive. Although relational integration between the doctors appeared high, there was little evidence of engagement with the practice nurse or practice manager in the intervention. There was some evidence of appraisal with both the practice manager and owner GP pleased with the performance improvements relative to peers.

### Case 5 (Fig. 6)

#### Mechanisms of implementation

There was strong overall coherence by all levels of staff in understanding the objectives of the intervention and its alignment with existing activities. This in turn fostered immediate action to engage with the intervention (cognitive participation). These actions and processes were variably implemented because management decided to not provide non-GP clinical staff with the tools due to concerns of it impacting on existing workloads. This impacted relational and contextual integration of the intervention into the organisation. Some GPs would have preferred AHWs to use the tools to engage patients as part of their existing role in performing frontline screening assessments prior to seeing the GPs. Despite this limitation, the HIO played a central role in cognitive participation, collective action and reflexive monitoring. Use of the site’s existing monitoring and evaluation platform assisted in communal appraisal. Regular team meetings were held where data and performance were reviewed with a specific focus on improving GP prescribing to the high CVD risk patient population. This likely played a key role in the increase in prescribing during the trial period.

### Case 6 (Fig. 7)

#### Mechanisms of implementation

All levels of staff appeared to have moderate understanding (coherence) of the intervention and its objectives. AHWs were enthusiastic about using the tool for screening and patient risk communication with support from lead GPs. Most staff used the patient risk communication graph component only and were less confident in using the other components. Many described time constraints in using the tools. In addition, information technology (IT) infrastructure and technical issues were major barriers at this site. This prevented long-term use of the intervention by all staff and greatly diminished any prospects of collective action. Only the lead GP remained enthusiastic in use of the tool over time; however, this required extensive time with the IT helpdesk to resolve software problems. Further, although this GP was the senior clinical lead, he lacked the authority to make managerial decision as this is the role of the chief executive officer. Another factor that inhibited collective action was a perception that the data extracted from the audit tool was unreliable, and this discouraged use by the PM and a newly employed GP. This also inhibited opportunities for reflexive monitoring despite the research team’s efforts to provide performance feedback reports and explain the reasons for data quality issues. This lack of collective action and reflexive monitoring are likely drivers in no change in the screening and prescribing outcomes.

## Discussion

This process evaluation of a multifaceted computer-guided QI trial in Australian primary healthcare settings sought to better understand why there was increased CVD risk factor screening and no effect on the prescribing rates in the intervention sites compared to the usual care during the trial. In depth examination of six case studies revealed a complex interaction between implementation processes and several contextual factors. The findings complement previous work highlighting the multiple barriers to uptake of health technologies into routine practice. These include knowledge-related barriers, sufficient training, specific features of the technologies themselves, the external environment, coherence for both providers and patients, and organisational context [35–38].

Despite knowledge of these barriers, the challenge remains in identifying strategies to overcome them. Given the diversity of the cases and the contextual circumstances in which they operated, there is clearly no one recipe for success or failure. The findings illustrate that there may be different factors at play during initial implementation compared to those that are needed to influence sustained use of the intervention. There appear to be spheres of influence that when aligned enhance normalisation of the intervention into routine practice. The first broadly relates to the mission of the site, its organisational culture and the antecedents to participating in this project. The second related to the leadership structures and the role of influential leaders in changing the activities of others. The third relates to the team environment and the extent to which certain actors within the team influence the activity of others. The fourth relates to the tools themselves and the degree to which they are fit-for-purpose from content, workflow and technical perspectives.

### Organisation mission and history

One of the ACCHSs (case 5) had prioritised the use of CQI processes over 10 years, and this was evident in strategy documents, staffing allocations and prior use of various CQI tools. Its high baseline performance is reflective of this commitment. However, the intervention was strategically determined by chief executive officer (CEO) to prioritise use of the tools by GPs only to the exclusion of other clinical staff. This supported improved performance in prescribing outcomes (the domain of GPs) and less movement in screening outcomes (the domain of nurses and AHWs). Such strategic choices were made explicit in this large organisation and could be linked to its policies and procedures around QI processes. In the smaller general practices, such strategic processes were less explicit but still played an important role in driving engagement with the intervention. For example, the teaching practice (case 4) had made as part of its mission a long-standing commitment to teaching excellence. Consequently, the intervention tools were avidly promoted to GP registrars as a part of this overall organisational commitment. NPT describes the alignment of the innovation and organisational mission as contextual integration, and in our cases, this was a driving factor [39].

### Leadership

A recent systematic review of the impact of clinical leadership on adoption of health information technologies found that the leader’s attributes and behaviours strongly influenced engagement [40] which supports Bodenheimer’s notion of ‘engaged leadership’ being the foundational building block of a high-performing primary care [41]. We found that the influence of leaders varied greatly. Although in case 1 there was strong motivation from the GP to improve CVD risk management practices, this alone appeared insufficient. Importantly, when the motivated leader’s interests were aligned with those of his trusted practice manager, then engagement in the new practice was enhanced. However, this also appeared to be insufficient, and when the support provided by the research team was removed, the intervention was used infrequently emphasising the importance of ongoing provider training. Cases 2 and 3 represented ‘one-person shows’ where utilisation of the intervention was entirely dependent on the GP owner. In the former case, utilisation was high and strongly driven by a desire to outperform peers. In the latter case, utilisation was low from the outset and over time came to be seen as a nuisance. In both cases, the intervention had little prospect of normalising across the practice once the study was completed. Curiously, however, in case 3 where the GP was least enthusiastic about the intervention, there were large sustained improvements in prescribing outcomes suggesting some behaviour change had occurred despite antipathy for the intervention. In case 6, although the GP leader was strongly engaged in some elements of the intervention and encouraged staff to use the intervention, he did not appear to take on a role of mentoring/training other staff. Further, organisational goals were more focused on individual patient care rather than CQI. This limited the impact of the audit and feedback and peer-ranked performance tools which are intended to make organisational performance more transparent.

While in case 5, the long history of leadership in CQI, supported by the governing board and CEO, influenced improvements in trial outcomes but it did not translate to normalisation of the intervention. This highlights the importance of ‘special people’ as key to successful implementation of HIT [42]. We found that these ‘special people’ include both clinical champions and non-clinical staff who have the ability to both broker and stifle engagement with an innovative practice. The findings suggest that when implementing QI interventions, it is important to identify and support ‘engaged leaders’ early to maximise potential for embedding new practices.

### Team work

Although teamwork is a key ingredient to enhance uptake of innovative practices [43, 44], the influence of teams manifested in complex ways in the case studies. There was no evidence of association between the team climate or job satisfaction scores and uptake of the intervention or trial outcomes. Indeed, in some cases where these scores were lower than average, performance was higher than average (case 5) and vice versa (case 3). This contrasts with previous studies which have shown that team climate scores are positively associated with staff satisfaction and improved quality of care [32, 45]. NPT conceives healthcare as a collective activity requiring a multitude of interactions between professionals, patients, managers and others. Rather than affecting only one individual or group, a ‘successful chain of interactions’ is required [46] such as leadership, strong managerial relations, readiness for change, a culture of staff training and resource availability [47]. Where teams are small and aligned (e.g. practice manager and solo GP in case 1), the chain of successful interactions may be less complex, making the work of integration less onerous. Conversely, in large teams (e.g. multidisciplinary care teams and several administrative staff in ACCHSs) with multiple roles, the intervention appeared less likely to influence staff interactions. Cases 5 and 6 (ACCHSs) had low ‘team vision’ and ‘task orientation scores.’ These measures relate to a shared sense of purpose, belief in the team objectives and reflective action on the outcomes that the innovation is generating. Even in case 4 (a general practice) where there was strong alignment of mission, leadership and shared purpose by the team, the high turnover of training GPs was an important barrier. Further, there was little engagement with practice nurses despite chronic disease screenings often being a core role for these staff. The intervention components were viewed mainly as GP and management tools rather than whole-of-practice tools.

It was clear from this study that support provided by the research team played a central role in driving engagement, and it is not surprising that there was a plateauing of trial outcomes in many cases once support was reduced in the post-trial period. A recent systematic review of decision support systems for prescribing highlighted that lack of training and limited computer skills were significant barriers to uptake [48]. In addition, several studies have found that the most effective training is tailored to specific provider’s needs [49], offers a variety of training formats and is provided on an ongoing basis [38, 50]. This suggests that there is an important role for external practice facilitators to reduce the work that insiders may have to do to support uptake [51]. In Australia, primary health networks [52] employ QI support officers to provide such a role; however, the degree to which they are accepted into practice processes is currently unknown.

### Tools that are fit-for-purpose

One appealing feature of the tools was their multifaceted nature targeting gaps at the system, provider and patient level [53]. In case 1, the tool components were synergistically incorporated into the practice with the manager taking ownership of the audit tool and the GP focusing on the in-consultation decision support tool. This facilitated initial adoption of the intervention; however, sustained engagement of the research team was required suggesting a lack of normalisation beyond the trial setting. Certain staff gravitated to features of the intervention (clinical managers using the audit tools and GPs using the decision support and risk communication tools) with lack of cohesiveness within the health service staff to integrate these features collectively. This prevented reinforcement of the value of the intervention to others in the health service. Although in majority of cases, the tools had high appeal in terms of content and usefulness, there were two cases (case 3 and case 6) where technical problems grossly impacted its use and led to early abandonment. In case 3, the low level of initial interest combined with frustrations that the tool was slowing software systems virtually eliminated any prospect of it being used (enrolment). In cases 2 and 6, it was only the high level of motivation by the lead GPs to solve the software installation problems that enabled sustained use over the trial period. In addition, time constraints and lack of financial incentives were a major issue in using the intervention during the trial and beyond. Participants from both cases 2 and 3 stated that financial incentives would have helped to sustain the use of the intervention.

### Strengths and limitations

Applying NPT in both the design of the process evaluation and coding framework provided a practical way to understand key activities involved individually and collectively in investing and enacting on the meaning, commitment, effort and appraisal of the intervention over time and across diverse primary healthcare settings. Our findings provide important insights into the interaction of context and mechanism (socio-technical change) to produce the resultant outcomes. It enabled us to systematically analyse a complex social and behavioural processes through several different ‘lenses’ moving beyond psychological theories of behaviour [54]. There are multitude of theories, models and frameworks to gain insight into how implementation of complex and multifaceted interventions can succeed beyond trial settings [55] and identify how these processes influenced the overall trial outcomes. NPT provided an explanatory focus through its emphasis on human agency [56]. By elucidating differences in implementation processes over time and between settings and various actors, we have been able to develop a nuanced understanding of intervention fidelity moving beyond whether it ‘worked’ or not.

A number of limitations need to be mentioned. The process evaluation was implemented toward the end of the trial, and while this was intentionally planned to not unduly influence conduct of the trial, providers may have limited recall of the intervention in its early stages. The cases studied clearly represent a limited snapshot of Australian primary healthcare, particularly given most general practices were located in an urban setting, two cases did not agree to participate due to time constraints and only selected providers were interviewed. Consequently, there may be other important phenomena that influence intervention normalisation in different settings that we did not observe. Further, by focussing mainly on staff, we were not able to fully appraise how the tools influenced the interactions between patients and health professionals (interactional workability). Although we intended to do a multilevel regression model analysis to assess associations between job satisfaction and team climate (as per our published study protocol), we did not identify any statistically significant associations on univariate analyses. Given the small number of participating sites, it is possible the study was underpowered to show a difference. Another important issue was that the lack of usage analytics (for reasons described in the methods) limited our ability to look more closely at adoption and fidelity measures. As part of the overall process evaluation, we conducted a video ethnography study and post-consultation patient interviews to provide insights into how the intervention tools were drawn upon in the clinical encounter. Initial discourse analysis has been published and further analyses are currently underway [57]. We also did not explore technical support staff perspectives which may have shed more light on the technical challenges encountered at some sites. Finally, resource constraints are likely to be major barriers to the ‘work’ done by staff members and we did not conduct detailed analyses of existing IT infrastructure, budget allocations to support use of IT tools and staffing allocations for quality improvements.

## Conclusion

This study evaluated the processes by which primary healthcare services engaged in a multifaceted computerised intervention. In doing so, we identified key mechanisms of why particular outcomes were observed highlighting the complex interaction of the tool and the environments in which they are implemented. These processes do not necessarily distil into a formula for successful uptake and improved outcomes. Rather, they may help to determine what trajectory a primary healthcare service is likely to take when engaging with such interventions. The findings of barriers to long-term adoption suggest that there needs to be sufficient lead time at the site to identify and act on any organisational changes that are needed prior to the intervention being implemented (e.g., governance, management processes, resource allocation, and staff roles and duties routines). An organisational mission that embraces quality improvement, engaged leadership and activation of all team members, dedicated quality improvement personnel, financial support, strong IT infrastructure and regular appraisal of outcomes are all key contextual enablers. Further, government payment reforms providing subsidies to using CVD risk assessment guidelines can raise perceived value to healthcare providers thus increase uptake of these types of tools. Greater appreciation of these factors can yield important information for intervention designers, academics, providers and policy makers to assist in adoption of computerised, quality improvement initiatives.

## Additional files

Additional file 1: Development of the intervention. (DOCX 13 kb)

Additional file 2: Adapted—Center TRT Evaluation Framework logic model. (DOC 92 kb)

Additional file 3: Health Professionals Interview Guide. (DOCX 34 kb)

Additional file 4: Context and outcomes of the cases. (DOCX 16 kb)

Additional file 5: Health professional interview findings and quotes. (DOCX 83 kb)

## Abbreviations

AA: Administrative assistant
ACCHS: Aboriginal Community Controlled Health Service
AHW: Aboriginal health worker
CEO: Chief executive officer
CQI: Continuous quality improvement
cRCT: Cluster randomised controlled trial
CVD: Cardiovascular disease
GP: General practitioner
HIO: Health information officer
HIT: Health information technology
IT: Information technology
MRC: Medical Research Council
NHMRC: National Health and Medical Research Council
NPT: Normalisation process theory
PM: Practice manager
PN: Practice nurse
QI: Quality improvement
TCI: Team climate inventory
TORPEDO: The Treatment of Cardiovascular Risk in Primary Care using Electronic Decision Support
