# Supplementary material for: What drives adoption of a computerised, multifaceted quality improvement intervention for cardiovascular disease management in primary healthcare settings? A mixed methods analysis using normalisation process theory
Source: Implement Sci. 2018 Nov 12;13:140. doi: 10.1186/s13012-018-0830-x (PMC6233504; doi:10.1186/s13012-018-0830-x)
Supplement: Supplementary file 1 — Development of the intervention. (DOCX 13 kb) [file 13012_2018_830_MOESM1_ESM.docx]

**Additional file 1. Development of the intervention**

| Validation testing | 11 primary healthcare services |
| --- | --- |
| Sociotechnical approach to the design | Integrating technology into practice requires human work to re-contextualise knowledge for different uses in complex social settings |
| Theoretical underpinning | - Lipsky’s concept of street-level bureaucrats:   Doctors means of expeditiously processing large workloads and how technological tools can influence the process.   - Gabbay and Le May’s concept of ‘mindlines”:   Professionally sanctioned codes of practice that may or may not relate to evidence-based guidelines. |
| Systematic review of clinical decision support system | Kawamoto et al. (2005) found that timing of advice when decision is being made was critical. |
