# Supplementary material for: What drives adoption of a computerised, multifaceted quality improvement intervention for cardiovascular disease management in primary healthcare settings? A mixed methods analysis using normalisation process theory
Source: Implement Sci. 2018 Nov 12;13:140. doi: 10.1186/s13012-018-0830-x (PMC6233504; doi:10.1186/s13012-018-0830-x)
Supplement: Supplementary file 2 — Adapted—Center TRT Evaluation Framework logic model. (DOC 92 kb) [file 13012_2018_830_MOESM2_ESM.doc]

**Problems**

- High prevalence of CVD
- Lack of screening for CVD risk & provision of recommended treatments
- Unaware of latest guidelines and recommendations

**Solutions**

- Multifaceted QI intervention
- Evidenced based approach on use of absolute risk
- Use of point of care electronic decision support system
- Use of audit and feedback tool for practice level improvements
- Engaging patients
- Training and support by experts

**Stakeholders/
Politics**

- PHC -GPs and ACCHSs
- Healthcare providers (HP)
- Administrative staff
- Patients and communities

**Other**

- Existing policies/ QI programs
- Administrative structures
- IT infrastructure
- Incentives for HP and patients
- Material resources
- Monitoring health services

**Continuous Engagement of Stakeholders, Intended Users**

**Outcomes/Effectiveness**

**3. Implementation**

TORPEDO Study (cRCT)

[Peiris et al. 2012; Peiris et al. 2015]

1. **Enactment**

- Engage stakeholders
- Enact plan
- Enacted plans and policies
- Type and number of resources leveraged
- **Reach** to health professionals and patients (intended population)
- **Adoption** by PHC (settings/sectors)
- **Implementation** as intended & acceptable/
  feasible/
  affordable

**4. Maintenance/
 Modification**

- Maintain relationship with PHC
- Monitor performance
- Ongoing training and support by experts
- Establish partners and vendors for implementation & evaluation
- Proposed plans

**Gather credible evidence**

1. **Formulation**

- Review evidence
- Pilot study (Peiris et al. 2009 & 2011)
- Modify QI intervention

**Inputs**

**Activities**

**Outputs**

**Data sources**

**Short Term (1-3 years)**

**Long Term Goal**

**(Public Health Impact)**

**(4-9 years)**

- Increase in CVD screening and prescribing
- Increase control of CVD risk factors
- Decrease in CVD incidence
- Improved quality of life
- Increase efficiency in the primary health care system

**Equitable** distribution of improvements across population subgroups, particularly those at greatest risk

**Minimal/ no unintended
consequences**

- - - - **Maintenance** of funding, partnerships, implementation, enforcement & reach

- Modifications for intervention and implementation based on Studies 1-4

**Primary outcome evaluation from cRCT**

10% absolute increase in recommended CVD risk screening. No improvement in prescription of recommended treatments overall

**Study 1:**

Plateauing of CVD risk factor screening performance in the 18 months post completion of the RCT. Ongoing improvement in prescribing performance in both control and intervention arm practices

**Study 2:**

Subject of this paper

**Study 3**

Doctor’s communication of CVD risk alone is not sufficient to engage patients. Rather, effective communication required skilled interactions on the part of both doctor and patient to enable meaningful use of risk communication tools

**Study 4**

Modelling intervention in PHN population demonstrated ICER <$50,000 threshold

**Disseminate & utilize findings**

**Justify conclusions**

**Study 2 – Quantitative/Qualitative**

Multi-level modeling/health professional interviews

**Study 3 – Qualitative**

Video-ethnography of actual clinical encounters and post- consultation patient interviews

**Study 4 – Quantitative/Qualitative**

Cost consideration of scale-up If implemented by an Australian Primary Health Network

**Study 1 – Quantitative**

Post cRCT effectiveness

Plateauing of CVD risk factor screening performance in the 18 months post completion of the cRCT. Ongoing improvement in prescribing performance in both control and intervention arm sites

**Study 2:**

Subject of this paper

**Study 3**

Doctor’s communication of CVD risk alone is not sufficient to engage patients. Rather, effective communication required skilled interactions on the part of both doctor and patient to enable meaningful use of risk communication tools

**Study 4**

Modelling intervention in PHN population demonstrated ICER <$50,000 threshold

Subject of this paper

Doctor’s communication of CVD risk alone is not sufficient to engage patients. Rather, effective communication required skilled interactions on the part of both doctor and patient to enable meaningful use of risk communication tools

Incremental costs of implementation <$15AUD per person.

If optimal effect sizes could be sustained over 5 years, the incremental cost per CVD event averted would be <$20,000AUD
