# Supplementary material for: What drives adoption of a computerised, multifaceted quality improvement intervention for cardiovascular disease management in primary healthcare settings? A mixed methods analysis using normalisation process theory
Source: Implement Sci. 2018 Nov 12;13:140. doi: 10.1186/s13012-018-0830-x (PMC6233504; doi:10.1186/s13012-018-0830-x)
Supplement: Supplementary file 3 — Health Professionals Interview Guide. (DOCX 34 kb) [file 13012_2018_830_MOESM3_ESM.docx]

# **Additional file 3: Health professional interview findings and quotes**

| **Case 1 – Small urban general practice**  Interview date: August 22, 2013; Time of interview: 11 am (practice manager (PM)) and 12:30 pm (general practitioner (GP)) | | | |
| --- | --- | --- | --- |
| **MECHANISM** | | | |
| **Coherence**  **(sense-making work)** | **Cognitive participation**  **(relationship work)** | **Collective action**  **(enacting work)** | **Reflexive monitoring**  **(Appraisal work)** |
| Differentiation  The main two staff (the GP and PM) both had a clear understanding of *HealthTracke*r (HT) and Clinical Audit Tool (CAT) and how it differs from existing practice. GP focused on HT and PM on CAT. PM encouraged GP to use HT and to populate data in correct fields within electronic health record (EHR) for CAT to be accurate.  Individual specification  After participation in webinar, GP had good overall understanding of the HT intervention and reason for use.  For PM, CAT could assist in data quality and helped other staff (i.e. nurse) with chronic disease management. She believes that audit tools help improves data quality and assess outstanding patient indicators. She is a promoter of audit tools.  **PM**: ….*he’s taking blood pressures all the time but what was happening he actually wasn’t putting in the right spot in Medical Director….by using that audit tool you can go “hold on”.*  Communal Specification  Both PM and GP collectively understood that value and benefits of the tool was to improve patients’ health and their knowledge of care processes.  Internalisation  GP and PM are motivated by  improving quality of care and  patient outcomes.  GP convinced more than before of treating early for high risk without cardiovascular (CVD).  PM believes that CAT helps entire health system inevitably helping patients attain better quality of care.  **GP**: *…focus on improving the*  *patients’ sort of health, and*  *improving my knowledge of it*  *as well.* ….*Greater service, a better*  *service to patients, especially*  *those with chronic health*  *problems and with CVD risk. I*  *think it’s really good.*  **Meaning**  **GP***: useful tool in the sense of knowing how to, sort of how to reduce the patients’ risks with cardiovascular disease and kidney disease and so on.* | Initiation  The PM is the main driver in helping coordinate implementation of intervention. PM is familiar with e-health and its benefits of improving patient care through data being linked with various e-health tools.  PM is familiar with the intricacies of all the GPs and staff work/habits and use of technology.  Legitimation  Both GP and PM found the study to be valuable to the practice in different aspects; GP got involved initially to improve CVD management/knowledge, and PM was sceptical. PM later was a believer in the intervention due to the substantial improvement in data quality at the health service.  **GP:** *I could see it was going to be beneficial for me and the practice. So, it was really good.*  Enrolment  GP used HT routinely for screening which became routine. Other components of HT were not used to their full potential due to unawareness and lack of confidence in tool.  Both GP and PM use their respective tools (HT and CAT). PM uses it to inform others of data quality and can see how it can affect data being uploaded to the hospital and benefit the patient. PM sees the bigger picture of use of e-health interventions.  PM is training one receptionist on why and how to use CAT. Other GPs are not familiar with HT. One part-time GP after several years recently started to use EHR and other GP is only involved in women’s health. PM suggested main GP to train part-time GPs.  PN could benefit from HT however not using it. She was not approached to participate in the study despite her main role taking care of chronic disease patients.  **GP***: …highlighted the issues that’s important in treating these patients, and so it’s becomes second nature now to know what to do and how to do it*.  Activation  With regular support and training for GP and PM, the intervention can be sustained. Need all staff to be involved to be able to be sustain completely and integrate into routine work.  **Commitment:**  **GP:** *the routine of checking for the microalbumin is something I didn’t do before, and now I realise its benefits.* | Interactional workability  HT aides in communicating CVD risk. Patients find graphs to be helpful and enlightening. The GP uses the heart-age projectile graph to communicate CVD risk to patients.  GP was not familiar and comfortable with all the features of HT and only used the traffic light assessment prompts and heart-age projection graph. He does not completely understand absolute risk % and therefore does not use to explain to patients. Further, he was not familiar with CAT or IF portal in relation to intervention. He did view the peer ranked graphs distributed to him by study team member.  **GP**: *Visual presentation of what they can do with it. It’s much easier to show them, you know, you can tell them, but to actually for them to see where they should be ideally, and then where they are at the moment, that does make them realise what a big difference that is there.*  Relational integration  PM assesses GPs’ data quality and outstanding CVD risk screening using CAT. She gives main GP feedback on his use of HT.  The intervention has made the GP and PM’s jobs more efficient.  **PM:** *It helps our nurse, so then we ah look, this person hasn’t had such and such for a while wo when you’re doing a chronic disease management say for cardiovascular or diabetes or anything like that, that all shows up as well. And so then we can then say to Dr. XXX (main GP) well look this hasn’t been done either because he might not have seen that patient in that last six months….so it’s like feedback from the nurse and also the doctor as well.*  Contextual integration  GP would prefer HT to be incorporated within MD. He finds it frustrating that it is separate. Also, SideBar is too large and must minimise the tool which causes GP to forget to open to use with patients.  GP had problems signing in and needed regular extra support. He would prefer someone to call to check in on how things are progressing with the intervention.  CAT would stop working occasionally. PM would have to call PEN support. Needed a reminder on using it and how to use it on a regular basis.  When tools were working properly, GP and PM found it to have integrated within their routine work.  **GP***: It’s not, it* [HT] *doesn’t really take any more time or effort; you get to the stage where you can use it without even thinking anymore*  **PM:** *they’ve* [patients] *come to the doctor and said look can you, you know, send a report up into the clouds, so if they end up in the hospital or the hospital can send things to us. But now you have that you need to have the right data in there, you need to make sure that the medications are up to date, their last blood pressure and you know, the pathology that you know, whatever their chronic condition is, it needs to be equalling whatever is in the system. So to be able to get rid of the old prescriptions out of there or the people who are no longer patients I wouldn’t have been able to find that any other way*.  Skill set workability  GP given the capability of the knowledge and reasoning behind the tool and its benefit by a ‘champion’ via webinar. This motivated GP and gave him confidence to use two components of HT: prompt (traffic light) and patient risk communication.  PM was proficient in using another audit tool therefore she had the skills and knowledge to learn CAT quickly.  GP and PM operationalised part of the intervention that was appropriate for their skills and role.  There is partial collective participation. Not all staff are involved and trained.  **GP:** *…eventually I’ll get to the stage where I’ll want to use them also for my resources and guidelines as well. It’s just a matter of time, just get used to it because, you know, just once I get the hang of it and it gets more useful I guess I just need to expand what I’m using, because I guess it can offer me so much more than what I’m using it for.*  **Effort**  **PM:** *I’m going to be running these lists and I know who’s going to be looking at these people and cleaning that data and it is a really, I mean it’s actually quite exciting when you do it. And I actually quite like doing that and I think if at the end of the day you’re helping that patient you know, that’s the best thing about it*. | Systematisation  CAT tool helped health service assess how they were performing, and if there were any issues with their data quality. The tool assisted the PM to cleanse data and identify any issues.  Individual appraisal  PM gives GP reassurance that he is performing well in screening his patients using HT.  **GP:** *seeing their cholesterol going down and their kidney functions improving, you know, that’s the real, yeah, when those results come through that makes me happy.*  Communal Appraisal  GP received reports of peer ranked graphs to see how they were performing compared to other health services. He found it beneficial to know how they were performing relative to other practices; however, doesn’t put too much emphasis on comparing themselves to other health services. Further, the report was not discussed with health service team members.  Reconfiguration  There was no reconfiguration of the health service.  **Comprehension**  **PM*:*** *having the patient coming in, that [HT] coming up and the doctor being able to better treat the patient would be, I would say, the main impact … referred if so to the nurse and obviously, she’s then got the data that the doctor’s put in and makes her life a bit easier as well... Overall caring of the patient I think, it really has improved* |

| **Case 2: Small urban general practice**  Date of interview: January 22^nd^ and 23^rd^ 2014 (main GP); January 30, 2014 (PM); January 31, 2014 (part-time GP) | | | |
| --- | --- | --- | --- |
| **MECHANISM** | | | |
| **Coherence (sense-making work)** | **Cognitive participation (relational work)** | **Collective action (enacting work)** | **Reflexive monitoring (appraisal work)** |
| Differentiation  Main GP and part-time GP see the benefits of using absolute risk and believe this is a better way for CVD prevention and management.  Both GPs saw the benefits of HT and it’s use with patients.  **Main GP**: *Prior to the Torpedo trial I used a fair bit of that* [online absolute risk calculation]. *Very similar in terms of what to do. But they are a bit more tedious. You got to punch in the information and so on. Whereas the HealthTracker can just extract the information much quicker. So those guidelines I look at more often. I haven’t looked at a lot of other cardiovascular guidelines...normal practice you might not consciously go in and do it. Being having a HealthTracker sitting there, giving you a prompt and say “You need to go look at it”.*  Individual specification  Data quality is important to the main GP. He saw the benefits and value of CAT for data cleansing. However, other staff were not involved and did not know what value it would add to the practice.  Main GP wanted to see how his practice was doing regarding CVD management compared to others as main reason for participation in the trial. Thought tool would be good platform to view how he was performing. This would assist in picking up additional patients that have incomplete screening and prescribing. Motivated by competition.  Both GPs recognized the benefits of using HT to improve patient care and data quality.  PM does not see the benefits or value of the intervention for the health service due to lack of interest and capacity to participate.  **Part time GP:** *it is something that* [absolute risk calculations] *we do as common practice, I do it in the other practice but it’s not the same, just that online one and we discuss it with a patient and do implicate whatever it is. So I do use, like to use it* [HT], *and I was really very excited.*  Communal Specification  Both GPs used absolute risk as method of managing CVD risk. They both saw value and benefits in HT tool for the assessment/care of their patients.  Part-time GP did not get involved in other parts of the intervention and was not aware of their objectives for the study.  Other staff were not made aware of the purpose of the intervention and study.  Internalisation  Main GP initially participated to improve health service patient data quality however realised the importance of HT in screening and prescribing according to guidelines.  **Meaning**:  **Main GP*:*** *there are certainly increased vigilance to screen the patient who is supposed to have the screening done. To look at their risk factors in a more comprehensive way because HealthTracker itself is look at it in a comprehensive, give you an assessment risk of a five-year period.* | Initiation  Main GP was the main driver of implementation of the intervention at the practice. GP has the knowledge and skills to be a ‘champion’ of the intervention for his staff and patients. However time and resource constraints prevented him from using it regularly.  Legitimation  GP liked HT and the risk communication component however only used for patients he thought of as high risk. Participation in the trial gave GP an incentive to use during trial period. HT allowed GP to become more vigilant in screening and prescribing according to guidelines.  Part-time GP uses other CVD risk tools on a regular basis and if this had worked for her, she would have incorporated into her daily work. She found the intervention to be novel and easy and beneficial for the care of the patients.  PM is not interested in any additional work besides what has been delegated to her due to time constraints.  Enrolment Part-time GP was excited about the prospect of using HT. Using absolute risk and risk projectile graphs is common practice for the GP at her other surgery. She was persistent in trying to use HT at different periods of the trial. However never could overcome technical issues.  Other staff not involved in the study due to time constraints. They did not seem to think it was something they should be concerned about with their roles and responsibilities. GP didn’t want to overburden them with other work.  Accreditation for all GPs or financial incentive for compulsory use of HT and CAT can help motivate the use of the intervention.  Main GP is more vigilant in prescribing after awareness of guidelines.  **Main GP***: belief that if not using HT, numbers [screening] would have dropped or stayed steady. Prompts are helpful*.  Activation  Main GP is the sole user of all components of intervention. Due to time constraints, he is unable to sustain long-term use of HT beyond the study. He needs more external incentives to get others at practice involved.  Main GP became more vigilant on using HT and CAT during the trial period; however, he believes there is more work that is needed to improve the intervention. Further, he needs allocated person to assist in implementation and maintenance of the intervention.  Screening and prescribing that have become somewhat routine for the main GP as a result in participation in the study.  **Main GP*:*** *I’d like to use it more. Unfortunately timing issues. But at the end of the day if there are some criteria that are set, that it becomes compulsory for accreditations, that could be a way of forcing all the doctors and the staff and the nurses, if you have one, to look at it at the regular fashion. And if you come out and say “Yes, the CAT tools are good” you’re supposed to be doing what is supposed to be doing, is good enough to be applied to 80% of the surgery for example, and we want at least 80% of the surgery using it to do it regularly, maybe at the three-monthly fashions, and extract the data,*  **Commitment:**  **Main GP:** *it strikes me as a good trial to get involved, to look at how robust my practice is, or was during the trial. And is not a big area analysis, it’s a small area of clinical practice. That’s why I think I could get involved. It’s only the cardiovascular side of it and it’s got an existing tool that you can use and you just see how we use it, how good it is and so on.* | Interactional workability  ‘What If” CVD risk graphs were helpful to  communicate to the patients their heart risk. Patient resources could have been better  if more concise and on one page. Patients were impressed with graphs and found resources useful when GP found time to use.  HT tool wasn’t used frequently by main GP because of time constraints. Further it was dependent on if patient were high risk or not.  Due to technical issues, part-time GP was not able to use HT. She uses similar patient risk communication tool online at her other practice.  **Main GP:** *Explaining to the patient, the graph is quite good. But I have to admit that I haven’t used it very often.*  **Main GP***: I’ve moved away from using single factors a few years back now. Is to use a combined risk factors. And it’s always championed to me that is the better way to analyse to the patient. But I find that they’re harder to explain to the patient with a number.*  Relational integration  There was lack of relational integration. GP is only staff using HT and CAT. GP has mentioned it to the PM however she is not keen on taking on any extra tasks outside her current role due to time and resource constraints.  There are no formal meetings on CQI programs or studies that practice is involved with. They have meetings only for big policy or system changes, otherwise meeting once a year on a weekend. A lot of corridor chat.  **Main GP:** *the administrative side of it needs improvement. If you ask the doctor to just go and look at it* [CAT] *himself every three months or so I think you’ll be hitting the wall. Okay. But if you get an allocated person to say “Well I’ve been allocated this task, and so every three months I do it* [review CAT]*.*  **Main GP***: A good single person allocated, keep monitoring, keep going, these tools will be very, very good. Yeah.*  Contextual integration  Ongoing technical issues with HT and CAT prevented integration of the tool at the health service. Part-time GP had the intentions to use the intervention; however ongoing issues discouraged use.  Main GP sees the value in HT however does not see it as a “robust” intervention. The intervention needs improvement. He would like it to be used quickly and efficiently. Time constraint and lack of resources is a factor.  **Part-time GP***: Every time I do the data on that* [HT], *it is just, it’s frozen up so I don’t think I have any input in their things. That’s what I you know, was a bit of, because I didn’t, you know I used it in the beginning but every time then it was a struggle and then we tried to fix it and it didn’t work, then I just stopped doing it….They* [Developers] *come but we tried it many times but then that’s it, we give up like after a while, I couldn’t, it’s always, it didn’t work*  ***PM:*** *I simply don’t have enough time because I have to do the recall while attending to the phone calls and receiving the patients. I don’t think it’s feasible. It wouldn’t be effective.*  Skill set workability  GPs have the skills and knowledge to promote the use of the intervention. Regular training/support over the phone for part-time GP and face  to face for main GP would encourage its use and provide confidence. Both GPs learn best by hands on training. Our training was sufficient initially.  Other staff were not trained on use of TORPEDO or any of the components of the intervention.  GP performed work from administrative, IT and regular patient care.  **Main GP**: *I haven’t allocated a task to my staff to use it* [intervention], *we haven’t trained the staff.*  **Effort**  ***Main GP:*** *It cannot be based on just me to do the analysis all the time, which is impractical at the end of it…fully integrated in terms of not just the doctors are using it, the staff are using it at the same time. So you need a bit of education for the staff, show them how to install it, how to log in, how to logout, where to get the information, which area they should be looking at, maybe even our administrative side of it, to help the doctors maybe.* | Systematisation  Main GP found peer ranked reports to be beneficial in assessing how he is doing compared to other GP practices. He used a few times during the year.  He prefers someone to come in and tell him how he is doing and where he is regarding the study indicators. Time constraint prevents him from analysing data on a regular basis, and lack of a dedicated staff member.  Individual appraisal  Data illumination from CAT and IF portal reports for main GP. He was motivated by data quality.  Part-time GP has reviewed reports that main GP has given her however does not motivate her because not aware of the bigger picture of the study. The reports informed the GP they are an average practice.  Main GP think HT helped improve their data.  **Main GP:** *I did tell someone halfway through that I was a bit embarrassed about our data of how many patients are supposed to have blood pressure pills and they’re not on blood pressure pill. And that I think there was another group, there was discussion and they say, I say “But after I look at all the other practices and I wasn’t feeling too bad, even though it was bad”. Even though the number was quite low, and I said that I’m not far difference.*  **Main GP**: …*others* [GP practices] *who improved. Improvement is a much better of course, so I know that yes, you can do better, by using all this Health Trackers and looking at the CAT tools and so on.*  Communal Appraisal  For communal appraisal at the practice, there needs to be regular formal meetings. Also, there needs to be an allocated person driving the monitoring and evaluation of the goals of the trial and intervention.  **Main GP***: get the person allocated to analyse and then feedback and say “Well we’ve extracted our data. This is the pictures. This is what it is” and you can just read that summary quickly and say “I need to concentrate on my, say, blood pressure documentation for this groups of patients”, or “My microalbumin has not been done for groups of patients and there’s too many that are not being done” and the data will come out with the summary. And that each three months or six months you would get a little bit of summary “I better do better in this area. Or this area’s missed out, or this area missed out”.*  Reconfiguration  Main GP worked with developers to fix problems so both GPs can use the intervention, however ongoing technical barriers deterred GPs from using or changing their practice to improve their data any more than it had.  Main GP would like intervention to be quicker, integrated within EHR and have an allocated staff to help drive the intervention at the practice to use long term.  No delegated person/point person to present ongoing reports of the study indicators (CAT and IF reports), trouble shooting of intervention with developers or study personnel, and organising training.  **Comprehension**  ***Main GP****: No, I don’t* [think] *it’s integrated very well at the moment, because one is I haven’t allocated a task to my staff to use it, we haven’t trained the staff to train once or twice.* |

| **Case 3: Small urban general practice**  Interview date: February 16, 2014; ~ 10:30-11:45 am (GP) and 12:15 pm (PM/receptionist) | | | |
| --- | --- | --- | --- |
| **MECHANISM** | | | |
| **Coherence (sense-making work)** | **Cognitive participation (relational work)** | **Collective action (enacting work)** | **Reflexive monitoring (appraisal work)** |
| Differentiation  GP has a personal interest in kidney disease. HT was a tool that was going help GP to be more aware of risk factors for diagnosis and treating kidney disease.  Prior to using HT tool GP did not use absolute risk calculations since it was too complicated and time consuming. Tried to assess CVD risk from individual risk factors. The tool assisted in assessing overall risk and kidney disease risk.  GP did not know the purpose for other components (audit tool and web-based portal) of the intervention; therefore, did not use. He used portal initially but did not see much point.  PM was not involved in the study therefore did not know the purpose.  **GP:** *we couldn’t complete that* [absolute risk calculation prior to HT]. *We could simply say to him, “You are in the bad department” …*  *…It* [absolute risk calculation] *was tedious, it was hard work. It still is like that but that gives us a bit more about absolute risk calculations.*  Individual Specification  GP lacks knowledge of the specifics and purpose of all components of the intervention. He is familiar with HT assessment and “what if’ graphs.  **GP***: HealthTracker made it more attractive because that showed that I was doing something and I was going somewhere, whereas all the others, just collect this, collect this, collect this.*  Communal Specification  The PM did not know purpose of the intervention or study. GP did not believe she was interested; and PM believes GP did not want PM involved.  Patients found it useful however GP not able to convince patients of changing their behaviour or medications for high CVD risk patients without CVD.  Internalisation  Saw overall benefits of using HT tool for cardiovascular patients and incorporating kidney screening. His personal influence of using a tool to screen for kidney disease was a major factor in its use.  **GP*:*** *I have prescribed for people to whom I can demonstrate that something is wrong and they need to fix it.*  **GP:** *it’s worthwhile knowing, however when it comes to treating people, they want to be looked upon as individuals and they want to be shown where they are going, not relative to somebody else*  **Meaning**  **GP*:*** *I could see that it was specific to most of my patients. Now, most of my patients had to do with heart disease and, of course, you could incorporate kidney disease as well in that.* | Initiation  GP is the solo driver of all programs and studies including patient care. GP works autonomously. He used HT tool for kidney disease and cardiovascular patients and those he suspected of being high CVD risk.  **GP:** *I began looking at everybody with kidney problems. I do lots and lots of blood tests.*  Legitimation  GP has a difficult time convincing high risk patients in taking medication. This is a regular occurrence and assumes patients wouldn’t be interested so doesn’t bother trying.  Further, he believes he a high performing site and didn’t need to change much after initial training from implementation team.  Enrolment  GP believes the concept of the tool is good; however, does not know how to use it properly. Needs incentive to use intervention to have impact, and needs further training.  **GP:** *difficult to convince patients to take the medication, although admittedly I understand the benefits of it* [prescribing to high risk patients]*.*  *…It’s a very good tool. It’s a very good system and I know it is developing because it’s looks newer, different each year and the use is just a matter of having to use it.*  Activation  GP’s method of patient care is face to face without focus on the computer. He uses computer to see if patients need further assessments and what was discussed previously.  GP set in his ways in patient care. He needs a lot of hand holding to become comfortable with new method of assessing CVD risk.  **GP***: It’s a good tool. It’s a good tool. If it is put to proper use it’s, the use will eventually serve the purpose for which it was built…*  …*now that I’ve used the thing for a little while, I’m aware, I’m conscious of its, of the management, of the use of it.*  **Commitment**  **GP:** *this one here [HT], bugger, it’s just a matter of collecting data. That’s how it feels, you see, so I haven’t got any, how shall I say it, enthusiasm about it.* | Interactional workability  At his practice, he wants to give his patients “total patient care”.  He initially used HT tool however went back to practicing the way he was prior to the implementation of the tool.  Doesn’t find the graphs helpful in explaining to patients their risk. The mathematics of is too complicated for the patients, unless patient shows interest.  **GP***: occasionally when a patient is a bit difficult to understand and difficult about realising that he has got a risk with cardiovascular disease, then I produce the file in the hope of convincing them they have to do something about it.*  … *“I’m quite happy with that. That’s a good sign. Thanks, Doctor. Thanks very much, you know, you’re the first one who showed me”. So, you know, things like that do happen. Out of the 10 patients that I would use that with, about seven of them would say that*.  Relational integration  PM finds the study interesting. She has seen the audit tool, and peer ranked reports when study research team visited the practice. She would like to learn and be involved but her assumption from what GP has said in past is that he doesn’t want her involved. GP excludes PM from anything related to software system.  GP thinks she is not “keen” on the study and stated he would ask her.  **PM:** *I think he doesn’t like me doing it, he wants to do it himself, that’s why. So I have no idea what he’s doing.*  **GP:** *Time factor is primarily that. You are right, if I could train xxxx(PM) to do it, I suppose she’d do it, but essentially, I didn’t know what it is for.*  Contextual integration  The tool slowed down his whole system. This caused him to dislike the intervention.  GP lacks time and resources to fully implement and embed the intervention.  **GP:** *Slows the damn thing down. It’s very annoying.*  Skill set workability  GP needs additional training and support to understand the rationale and use of the intervention. He found the initial training to be overwhelming. Incremental training and delegated time flagged explicitly with administrative assistant.  Doesn’t feel comfortable using absolute risk to prescribe. Needs more training and skill development. Age is a barrier in using health information technology  **GP***: I wasn’t paying 100% attention to that* [study/intervention training] *because these were just not appointment type attendances. Somebody just blew in and began talking about this* [intervention] *and I, so my patients are used to waiting but I don’t like them waiting*.  …*you’re really given a utensil and you don’t know too much about how to use it.*  **Effort**  ***GP:*** *“Oh, I’m all right. I’m doing okay so all I have to do is just carry on doing what I’m doing”.* | Systematisation  GP did not know how to use and read the peer ranked graph provided by the study team or use the web-based portal. He needed additional training. Therefore, there was no measure of health service performance of the study indicators.  There are no systematic method or practice of identifying benefits or issues with the intervention.  Individual appraisal  There was lack of interest in assessing how the intervention was impacting his overall performance in CVD management. He was happy with his initial performance of being above average compared to other general practices.  **GP:** *I was more keen on this fact that I was doing almost as well, then I wasn’t paying too much attention to the later graphs that came.*  **GP:** *Well, looking at this* [performance on web portal], *for example, where the red one is, it applies to me. I’d rather be on the other end like I did in the first time, like this one*. …*I suppose I didn’t know how to change that*.  Communal appraisal  Participants including patients did not appraise the intervention and its value. They gave some positive feedback initially when GP was using the ‘What If” graphs however impact was unknown.  Reconfiguration  There was nothing done to accommodate the intervention. Financial incentive would have helped use of the intervention.  There was lack of reflexive monitoring at the practice.  Lack of understanding of the study and intervention. Initial purpose to be involved was to assess kidney disease more efficiently.  **Comprehension**  **GP**: *it’s a bit of a nuisance and apart from the fact that it is beneficial in terms of putting figures down and calculating it, it appears as if I’m working for somebody else with no compensation and no recompense and it’s very annoying sometimes, especially when the thing clogs my computer now.* |

| **Case 4: Medium rural general practice**  Date of interview: July 17, 2014; time of interview: ~11 am (PM) and ~12 pm (GP) | | | |
| --- | --- | --- | --- |
| **Mechanism** | | | |
| **Coherence (sense making)** | **Cognitive participation (relationship work)** | **Collective action (enacting work)** | **Reflexive monitoring (appraisal work)** |
| Differentiation  Benefits of using HT as CVD risk management and teaching tool. Provided evidence for teaching registrars.  Main GP sees the benefits of having the best quality improvement tools at his health service however using the e-health tools has its obstacles.  **Main GP***: was using it* [HT] *as a risk measurement tool and as a patient teaching tool that people would, we would, a registrar would say “Gee I’ve just seen somebody who’s come in, who’s new, and have got a cholesterol of this and a blood pressure of this, and their sugar’s a bit high but they claim they’re not diabetic. What do I do?” and instantly we’d use it to say “Okay, let’s have a look at the risk. Let’s see what things we can modify. What can we do straightaway? What can we do down the track? What’s what? What can be, which order do you do it in” and so on. And at times what’s the evidence for it? Use it to teach wider range.*  Individual Specification  Due to the practice having mainly registrars with the owner being the main full time GP, it was a good way to train and teach doctors on best practice based on evidence-based practice, and use of new technology to enhance patient care.  Main GP values prompts to help screen and manage CVD and chronic diseases.  PM understood what the study was about however was not engaged in it due to time constraints.  Communal Specification  Main GP works on being the ‘clinical champion’ during team meetings. GP is an expert on diabetes management, and works at a diabetic clinic.  Other GP registrars saw the benefits of HT; however unaware of the frequency of use. Due to staff turnover, interest and awareness of the intervention was unknown.  Internalisation  Main GP valued it as a good platform to teach registrars and use to communicate risk to patients.  **Meaning**  **Main GP**: *I’ve been looking for non-paper based prompts for cardiovascular or chronic illness prevention. And that’s one reason. And secondly because I thought it’s something which was good to be measured. And thirdly I just wanted to see maybe overall how we were performing.* | Initiation  In order for all GPs to use HT, main GP needs to be a ‘clinical champion’ for using intervention.  Main GP dislikes computers therefore didn’t use as often as he may have wanted to and did not know the full capabilities of HT.  Main GP used in meetings as teaching tool and had research team attend meetings to train all the GPs. Registrars were keen to use.  **Main GP**: *..my age range is against me. It was, I’m not someone who’s intuitively familiar. So I attempt to use* [HT], *as I say I really hate computers…I tend not to sort of sit and say “Ooh I wonder what this will do” or “I wonder if I can find out this? I wonder if I can find out that?”….therefore if that’s my approach then it’s difficult for me to pass that on to other people* [GPs]. *I can’t sort of say go and do this [use HT] when I don’t usually do it [use HT].*  Legitimation  Main GP finds the tool to be beneficial but does not use regularly due to his confidence in computer tools. He needs additional training for him to promote it within his health service.  **PM***: I think the younger doctors found it* [HT] *useful, so that made things, the pop up reminders, “Okay, this person needs this checked, this person needs that checked”, that was useful for them.*  Enrolment  Theoretical concept was great however lack of confidence and knowledge with computer affected use of tool. Young keen and proactive doctors would be more apt to use.  PM not involved in the study and did not use CAT component for the study. PN could have been better able to assist with CAT.  Activation  Young doctors would likely be proactive and use HT; however due to staff turnover (registrars), use of HT was variable at the practice.  Main GP was less likely to use HT unless he felt confident in the rationale and knowledge of using it properly.  **Commitment**  **Main GP:** *you had a tool* [HT] *where you could instantly see what an evidence base risk calculator shows and you were able to modify it. Right, okay. And that was some of the other features like bringing up the feedback online and so on, “Let’s do this, remember the password, do this, it’s the internet’s slow. Bugger it. I’m going to go have lunch”.* | Interactional workability  For the main GP, the - ‘What-if” graph – he only showed the graph and didn’t manipulate since didn’t feel comfortable with explaining rationale. The graph was engaging for the patients.  **Main GP*:*** *People are interested. It’s a mode of engaging people.*  Relational integration  Practice has good staff rapport. Have regular weekly meetings as a teaching and team-building forum. HT was used to teach about difficult cases and its use with patients.  **Main GP**...*often will use it in case discussions in teaching that use it to actually say “Look, it’s there. You can use it and you can calculate and you can use it as a teaching tool for the patient, and you can use it as a prompt for yourself, what you can modify*”.  Contextual integration  Uses regular weekly team meetings to standardise practice and ‘continuing medical education’. Both administrative and clinical staff are invited to the meeting where both areas will be discussed.  The need of permanent GP staff could enhance the use of the intervention. Main GP was an advocate for the use of PCHR however doctor’s ineptness for computer was affecting its use. Young registrar soon to be permanent doctor had tips for using it more efficiently. Main GP was impressed and learned from young GP.  Three permanent GPs to join health service which could increase engagement in the intervention.  Non-GP staff were not involved and not interested. This would have assisted in overall use of the intervention and integration.  Main GP needs incremental training for him to use the tool regularly.  Skill set workability  Further training for main GP, PM and PN needed. Incremental training and once a month phone call for support would enhance use of the intervention. Possibly an online training demonstration could help.  High staff turnover is a cause of lack of collective action.  **GP***: the sole barrier was familiarity.*  **Effort**  **Main GP***: I remember you and xxx* [chief investigator] *would come around and you’d explain things and there would be things that I would see the benefit of, and then it was like “Okay, I’ve learned 25 new things. I remember three very well, I know how to use five, and I know I can sort of do this but I don’t quite remember how to do it”, and then after three weeks you’d think “Was there something like that there or not?” And you thought “Okay, I can go for a walk or I can sit here for half an hour and work out how to do it. I’ll go for a walk”.* | Systematisation  Used IF peer ranked reports to view the practice’s performance. Values feedback about how service is doing regarding screening and management of patients.  Individual appraisal  Peer ranked reports that identify progress needs to be summarised concisely via email in order for main GP to view the report.  **Main GP***: it was most likely once again, having a very, very short simple summary* [IF report]. *This kind of reporting is good where you sit down and have the introspection.*  Communal Appraisal  PM can add reviewing peer ranked reports at the Thursday meetings on a regular basis. This was done a few times however not regularly. PM has shown/given the reports to the Main GP.  Reconfiguration  There has been minimal alteration in the health service to ensure regular appraisal and use of the intervention. This occurred a few times during team meetings.  **Main GP:** *…use of the tools was increased obviously. Right, okay. But my use, or my way of using the guidelines really didn’t change.*  **Comprehension**  **Main GP:** *It’s integrated well, and there needs to be still more work. Look it’s a tool, and it’s a tool which has very, very many uses, and quite a lot of potential.* |

| **Case 5: Large remote Aboriginal Community Controlled Health Service (ACCHS)**  Date of interview: May 28-29, 2013; Time: ~10 am (GP); after lunch (Aboriginal Health Worker (AHW)); May 29^th^ at 10 am (health information officer (HIO)) | | | |
| --- | --- | --- | --- |
| **Mechanism** | | | |
| **Coherence (sense making)** | **Cognitive participation (relationship work)** | **Collective action (enacting work)** | **Reflexive monitoring (appraisal work)** |
| Differentiation  Staff that were introduced to the intervention saw the benefits of using the point of care component of the intervention. They saw the benefits of having easy access to the CVD risk screening profile and absolute risk displayed with ‘one click’.  The screening team and GPs had already been using the Doctor’s Control Panel (DCP) however the tool lacked calculation of absolute risk and patient risk communication. It was more of a screening tool. Therefore, they were keen on using HT.  **AHW**: *it is just the way it tabulates so much more information than what our other options have been. So again, it gives us a real accurate point of where they are with their risk assessments.*  Individual Specification  GP was an advocate in calculating absolute CVD risk, and prescribing according to their risk. HT made GP aware of prescribing guidelines and gave him confidence to prescribe “early”. Further GP had professional development motives for using the audit tool and absolute risk calculations.  **AHW:** *I liked it where we could show the predictions if they’d changed this or done that. Even though I know that doctors would go through that, I think also having an indigenous person as a health worker who only help maybe on that cultural side to say, well this is what this means, and it will be really handy in screening ‘cause not all our patients want to go through to a doctor.*  Communal Specification  GP was an advocate of using guidelines. He took on the responsibility of sharing guidelines with other GPs by making multiple photocopies.  AHW thought the ‘what-if’ graphs would be valuable in giving patients an Indigenous perspective.  **HIO**: *we do conduct business using absolute cardiovascular risk as our means to determine, you know, who’s looked after by what team*  Internalisation  HT provided evidence based medicine for CVD management which was a key reason for participation in the study. Everyone introduced to the intervention at the start really believed it would be beneficial to the health service.  **HIO:** *I think it’s a very good tool to have added to our kit. The fact that it’s electronic, the fact that it integrates with the medical record, I certainly see its value and purpose. I think the doctors certainly see its value and it’s, you know, sort of streamlined their consultations from the what do I need to do point of view, and then extended their consults on the how to engage with a patient*  **Meaning**  **GP***: It's helped my practice individually because I can get an accurate complex summary that takes into account all these additional factors and other relevant history, all the numbers, it gives me the reassurance that I'm getting an accurate cardiovascular risk, the most accurate really that I've seen by a long way. So it gives me confidence to prescribe the medicines that I prescribe knowing that my cardiovascular risk is accurate*. | Initiation  HIO worked exclusively on continuous quality improvement (CQI) programs and health system reports. She was the delegated staff working on TORPEDO and driving the implementation of the tool.  GP found the tool to be valuable and when possible, GP registrar would demonstrate the HT tool to other GPs. GP promoted and educated about the tool to colleagues. He would like AHWs who are at the frontline screening patients to use the HT tool.  Legitimation  HIO reviewed TORPEDO data on a regular basis (1-2 times per month). However, after trial completion, she did not review data as frequently. Driven by trial participation.  HIO used audit tool more often than peer-ranked web portal. Web portal was used to give an overview of the health service.  AHW was keen on using HT with his patients. He screens chronic disease patients before they see the GP, so it would have been valuable for him to show the visual presentation to the patient.  **AHW:** *I think as I work in that clinical sort of direct patients and really working close with doctors and all that, it could be a great tool for me to use. Someone in the community might be, it’d still be good for them but they wouldn’t have that, I think the visual tool, and that’s probably what I’m looking is that it’s just a great visual tool for me to use with my patients*  Enrolment  GP used tool to help reassure himself of his diagnosis and risk calculation, and management of high CVD risk patients. GP would buy the tool if he owned his own practice.  AHW wanted to use the tool; however never given access. He was not aware of reason for not being able to have access. He was disappointed.  **GP:** *it did change my prescribing criteria and having the Health Tracker tool available to help me calculate that reassured me that I was doing what is perceived to be the right thing.*  **AHW:** *it was interesting though that we were still shown it [intervention], and, so, you know. So you sort of, look we’ve got this great tool here, but guess what, you can’t use it. It was sort, that’s what it felt like to me.*  Activation  The intervention can become embedded and integrated if non-GP staff are given access to HT. And regular support and training is offered to GPs and non-GP staff.  **Commitment**  **GP:** *if I was running my own practice I'd pay for this software even if I was charged to use it and I'd use it often.* | Interactional workability  Patients appreciate the ‘what-if’ graph and being able to see their risk visually. GP used infrequently due to time constraints. He thought it was valuable component. Time constraint is a big barrier at the health service for GPs.  **GP:** *if you're going to go through all this you actually, you've got to be prepared to have a good 10 minute chat with the patient because you actually want to engage them and help them understand where they're at and make a difference and that's the time. So it's not the program time it's actually alright we're going to have a proper chat today…..that's what takes the time. So to just have that chat without this tool would, you know you'd be drawing all sorts of pictures over the paper and the patient might get the point but not really. But with this tool if you've got the time you can really get your message across. Yeah but it's that, sort of that 10 minutes to have a proper chat with the patient that I haven't, yeah.*  Relational integration  HIO worked with GPs to install HT, set up training and report on performance at meetings.  There was lack of use of HT across different roles despite interests. If non-GP staff used tool, it would be beneficial to GPs and overall patient outcome.  There are multiple care teams that work with different patient cohorts. There was lack of implementation of the intervention within these groups.  There was staff turnover that affected use of the intervention with new GPs.  Contextual integration  GP would like to see HT move information from HT into the EHR.  There was an issue with the resource and time constraints.  It would be valuable, and increase capacity for change if non-GP staff used HT tool.  **GP*:*** *it's like a one way street and doesn't come back. And so if I wanted to get any of these nice documents into my notes I don't really know how to do it apart from print it out then type it all in. And I'm not going to do that.*  **AHW***: I don’t know whether from management and whether they thought it was for the safety sake, you know, patient’s safety and the safety of us workers, you know, maybe too much stuff I think. But as we’re involved clinically I think it’s still, it’d be great to have access* [HT].  The ACCHS has a strong foundation of CQI programs. Our intervention fit in with other already integrated programs.  Skill set workability  Skill set depends on age of the doctor. Young doctors are more ‘IT savvy’.  It would be beneficial to have follow up calls and training session within 2 weeks of initial training. Follow up training and support is crucial in helping GPs use HT.  HIO, delegated CQI staff had experience using the audit tool and found web-portal as an important avenue of relaying information to the GPs.  **HIO***: I think it’s just about that training. So, and then following up, you know, so for example, the doctor has training, you know, following up a month later just do we need not a re-training but, you know, any questions. What we find is most of the doctors when they go to the initial training session they’ve, you know, they may not even have a login so they’ve never actually seen Health Tracker at all…*. they really benefit from having maybe a following up session. But, yeah, sometimes I feel that, you know, the time delays are really too far gone and they actually need complete re-training again.  **Effort**  **GP:** *It's got a big potential to help the practice but you've got to have people willing to use the computers and have the time in the consultation to go through it with the patient and unfortunately that hasn't been the situation in the last six to nine months at xxxx* [ACCHS name] | Systematisation  HIO would report to GPs their performance in TOPREOD especially prescribing to high risk patients quarterly at the GP meetings. She would have lists of patients that were not properly managed. GP were illuminated by the reports from HIO. They were keen to review patients that were suboptimally treated.  Individual appraisal  GP suggested providing all GPs with reports of performance of study indicators by point person, and then reviewing the data with GPs to motivate use of HT. This will demonstrate the benefits of using absolute risk.  At quarterly meetings, HIO presents data from CAT and IF portal on performance of all study indicators. GPs are very receptive.  Communal appraisal  Health service’s focus is on seeing how they are doing amongst themselves. Less concerned with comparing themselves with others. Focus in improving patient outcomes and quality.  **HIO:** *will extract the information out and then put it into our own, you know, xxxx* [ACCHS name] *looking report, and then report that back to the staff. Because only the GPs here use that we usually tie it into the GP meeting, so they’re held quarterly and we might not put TORPEDO on every quarter, but maybe, you know, it’s six monthly to allow for, you know, a bit more, so certainly I think, yeah, they’ve had at least two to three reports it must be, handed back to the GPs.*  Reconfiguration  HIO incorporated reporting on CVD risk factor indicators at GP meeting. HIO worked to check regularly that training was provided to new staff.  **GP*:*** *I've seen these graphs* [peer ranked web portal reports] *before in presentations, when presentations have been given, but I've never accessed it myself.*  **Comprehension**  **HIO**: …*for example only 30% of the high risk patients are being prescribed with triple therapy and they go, whoa, you know. So they really like that, and we can then actually provide them with the names of people, you know, and then sort of next time they present to the service they can have a quick look at their, you know, medication management.* |

| **Case 6: Large urban Aboriginal Community Controlled Health Service (two locations)**  Date of interview: November 25^th^-27^th^ 2013 and February 6, 2014 (3 GPs, 2 AHWs, PM, and practice nurse (PN)) | | | |
| --- | --- | --- | --- |
| **Mechanism** | | | |
| **Coherence (sense making)** | **Cognitive participation (relationship work)** | **Collective action (enacting work)** | **Reflexive monitoring (appraisal work)** |
| Differentiation  GPs, PN, AHWs, and PMs, saw the benefits of the intervention and impact it could have on patients with one click.  **Lead GP***: you’ve just got an independent source and it’s new data so it’s just refreshing your minds because the guidelines are always changing, the targets are always changing, blood pressure targets, lipid targets, so I, yeah, just brings everything, gives you a fresh review of everything, and it’s on your computer, it’s on your desktop.*  **AHW***: Well, usually when a patient goes in for a consult, they don't usually see anything like this. They might get a bit of paper with their care plan on it that's written in medical jargon and they don't understand it. When you've got something as basic and straightforward as that, it's an easy to read tool for anyone.*  Individual specification  Lead GP was sceptical at first due to technical nature; however, he is an advocate for managing patients based on absolute risk. HT provided a simple explanation for patients through the ‘what-if’ graphs.  All GPs gained knowledge and skills on screening and prescribing based on absolute risk  PM, PN and two AHWs believed that HT would be beneficial for their patient population at the HS. They were excited about the prospect of using the risk communication component.  **PM***: Initially, when we first got the side bar* [HT], *I was really impressed, and I did like the fact that it reminded you that things needed to be done, or not that needed to be done, but things that hadn’t been done that perhaps could be done, and I found that really helpful*.  ***GP3****: I think it’s* [intervention*] definitely made an impact. It’s helped me with my clinical practice. It saves me having to manually input all the things into the calculator, the Framingham risk calculator, and it’s a good reminder tool.*  Communal Specification  All staff using intervention found risk communication ‘what if’ graphs to have the most impact on patient outcomes.  Internalisation  GP and non-GP staff see the overall value in intervention being a preventive care tool.  **AHW***: It saves you heaps of time because in a blink of an eye you can see what they're due and what they're not, whether or not it could be save a load of time and provide a better care for the patient, making sure we're not missing anything.*  **GP2:** *Torpedo, as I said, when I see those graphs that I’m sharing with the patients, yes, for example I change, I change the medication like blood pressure medication from one level to two more levels, like three combination medications instead of just giving one. So it’s still one tablet, but three medications in it to improve the blood pressure.*  **Meaning**  **Lead GP**: *it sets the bar high and it does, it’s a great programme and I guess the constraints are just the time constraints. But it’s something that you need to look at, you know, if you’re going to do a good general practice and you’re going to do primary prevention you’ve got to use something like this…using something like this it’s got to be part of your practice otherwise you’re just doing reactive medication.* | Initiation  PM worked with research team to install HT and scheduling training; however, no delegated person for ongoing training or trouble shooting.  Lead GP and GP2 were an advocate of AHWs and nurses using HT as part of their screening practice. However, GP3 does not think others should have access to HT only because this will cause more time constraints.  **AHW***: I've been there a couple of times with GP x [lead GP] doing this* [‘what if’ graphs]. *And he's entered in the data. And then it showed on the projection. What would they do if they changed this, you know, change their smoking and then change that and then change this and watch it come down and see the patient's reaction to that? And even that for me, 'cause I'm a smoker [laughs], yeah, and there was a considerable jump in the risk when he just took away the smoking. So, yeah, it was a bit of an eye-opener. And it's pretty easy to read graph*  Legitimation  All staff believe that this intervention can improve quality of care. However many are not aware of the full capabilities of the intervention.  **Lead GP:** *routinely I would use it* [HT] *when I’ve done some, I’ve done a health check and I’ve done their bloods and they’re coming back and they’re looking at their lipid results, and people get a bit fixated on why is that number up, why is that number down or whatever, “Do I need to fix that?” And so you give them a holistic view of cardiovascular risk.*  Enrolment  All staff trained found the ‘what-if’ graphs to be powerful and engage patients. It was easier to explain to patients their CVD risk in pictures. They did not use often due to time constraints.  Activation  One GP and AHWs stopped using HT after it disappeared one day. AHWs were too busy to follow up. They were using for screening purposes.  Lead GP was more computer savvy and proactive in trying to fix issues on his own or call/email research team. However worked mostly autonomously due to his personality.  **Commitment**  **Lead GP***: I’d still go looking for it* [when HT disappeared]. *You know, the benefits outweigh the problems…I’m very much an advocate of it.* | Interactional workability  The ‘what-if’ graphs have impacted positively in helping patients understand their CVD risk profile.  Engaging and easy for clinical and non-clinical staff to explain graphs.  **Lead GP:** *you can look at the specific risk factors and what you can do about it, and see how you can change your risk. And so I like using, in that instance I love using the graph, I love using the change, the smoking status, to look at how you can change your heart age, and I’ll say, “If we put you on a statin it will drop your cholesterol from 6.9 to 4, and here’s what happens to your cardiovascular risk; your heart age drops to this, or it has relatively no change.” So yeah, just looking at the what-ifs.*  **GP2:** *pictures make more for impact than the words, kind of thing. So they were more affected by that. And some of them made the decision to, you know, to stop smoking, for example. And yeah, so that was a good effect.*  **…***Advantages, definitely showing the patient on a graph* [What if graph] *is effective. Because it’s a picture, they can’t, it’s clear, it’s not complicated, the graph. So you can actually see with a simple explanation and it’s effective. It’s impressive.*  Relational integration  Non-GP staff were provided assess and training to HT. Those screening patients used HT till it disappeared. Main GP and GP2 an advocate for AHWs to use HT for CVD assessment.  GP3: *At the moment there’s no one doing that role* [CQI officer], *so everyone’s just busy seeing patients and no one’s really looking into quality improvement.*  Contextual integration  Problems with audit tool not extracting data from EHR accurately. This discouraged GP3 and PM/PN from using for data quality purposes. PN used CAT frequently for audit purposes and study reporting. Due to working across two locations, there were issues with data accuracy.  They were unable to resolve the issue  Most staff did not use the intervention to its full capabilities. There was lack of knowledge of what comprised TORPEDO components.  Team meeting on a regular basis with focus on case conferences.  **Lead GP***: advantages are it’s on the side of the screen and it’s interacting with your software…*  … *disadvantages ..it did take a little while to load if you didn’t have it running already, and just a few software glitches, not with your programme but mainly with Medical Director, because most of the time the questions about this come up during a health assessment, Aboriginal and Islander health assessment which is a horrible programme in Medical Director that runs alone and you can’t move from it to any other programme. So you’ve almost got to hand write your little notes and then come back to at the end, and you always run out of time. So that’s just an unfortunate part of Medical Director, but no, that’s about all.*  **AHW: J***ust because of some discrepancies of where it's pulling information from, it's really let itself down. I mean, it saves you using the assessment tool. It saves you heaps of time because in a blink of an eye you can see what they're due and what they're not, whether or not it could be save a load of time and provide a better care for the patient, making sure we're not missing anything, you know.*  **Lead GP:** *then when the print status came up that was good because I could give people a hard copy to take with them.*  **GP3:** *It didn’t pick up all the patients, so for example say in April of last year I tried to see how many Aboriginal patients we saw for that entire month. And it came up with a number of about 20 or 30 something, which is not right because I had to, I went through manually all the patients for that month, and it turned out to be about 200. So it was not picking up all the patients. I don’t know why that’s happening. But I couldn’t use the clinical audit tool to verify my numbers, because it’s underestimated by a lot.*  Skill set workability  Main GP trained GP2; didn’t provide in-depth training. Newer GP did not receive formal training. He was given training over the phone by webinar.  Need for additional follow up training and support on regular basis. Computer literacy is variable.  100% staff turnover for AHWs during middle of the trial. Lack of transfer of training and knowledge about HT and study.  **Main GP:** *I mean I think you guys came around and did a few live tutorials, and that was helpful, and especially when new doctors came on board they could hear it from someone else; they don’t want to hear it everything from me**. I’m not very good at explaining things, just ask my kids.*  **Effort**  **Main GP:** *focused on case conferences and individual cases, and we don’t so much look at public health. … not a lot of remuneration for it immediately, so maybe we’re chasing the cash cow a little bit that way rather than stepping back and getting a bit academic.* | Systematisation  PN/PM working on data quality and clinical audits. PN works at new smaller location. This affects communication of data quality results.  Main GP sees value in CQI programs but no incentives to participate. Patient care is focus.  Individual appraisal  There was partial self-monitoring on performance after using intervention. Staff would see benefits if there was an impact on patient lifestyle after viewing ‘what if’ graphs.  **PM:** *Find these* **[peer-ranked reports from web portal]** *very valuable in looking at how we’re going compared to other services, and whether or not we’re actually getting to the potential risk factors in the patients that we’re seeing.*  Communal appraisal  Main GP/CEO would like a delegated point person, preferably GP, to report on study progress at team meetings.  New GP working on CKD audit, and used this as educational session on UACR screening and kidney disease.  **PM*:*** *I’m just really, really pleased that you’re continuing for another 12 months, because I think for the first six months of the project no one got it right, and I will be even more interested to see how much improvement we’ve made at the end of the next 12 months.*  Reconfiguration  CEO requested one of the GPs to focus on CQI once a week which can enhance intervention use. Need regular reporting of performance of TOPREDO study indicators at meetings.  **Lead G**P: *[GP3] got a whole day a week that he’s supposed to do this [data review extracted from audit tool], then that’s the place because I don’t really know what he does on most days, and I occasionally ask him and then I drift off when he tells me. So I think that’s, you’ve got him being paid one a day a week, you know, that’s where it should happen.*  **Comprehension**  **AHW2***: there was no follow up with it* [intervention] *within xxxx* [ACCHS] *itself to say, ‘How’s everyone going with this?’ ‘Are you using it?’ like, ‘Was it useful?’ and stuff like that. There was nothing.* |
