# Supplementary material for: What drives adoption of a computerised, multifaceted quality improvement intervention for cardiovascular disease management in primary healthcare settings? A mixed methods analysis using normalisation process theory
Source: Implement Sci. 2018 Nov 12;13:140. doi: 10.1186/s13012-018-0830-x (PMC6233504; doi:10.1186/s13012-018-0830-x)
Supplement: Supplementary file 4 — Context and outcomes of the cases. (DOCX 16 kb) [file 13012_2018_830_MOESM4_ESM.docx]

Additional file 4. Context and outcomes of the cases

**Case 1**

| - A small, urban general practice in a socioeconomically disadvantaged region of Western Sydney. - Staff members were the full-time GP owner and a practice manager (PM) who had both worked there for over 30 years with part time clinical and administrative staff. - The mean TCI score was above average when compared to the mean of all cases, however job satisfaction score was lower than other cases. - The practice was strongly dependent on the research team to stay engaged with the intervention. - There was approximately 10 hours of training and technical support provided during the trial phase and negligible support provided in the post-trial phase. There were multiple visits to the practice by the project officer to assist both the PM and GP but little dedicated training for any of the other staff. - Screening and prescribing outcomes significantly improved over the trial period but then plateaued with a slight decrease in prescribing in the post-trial period. |
| --- |

**Case 2.**

| - A small urban, residential Sydney general practice with a full-time GP owner, several part-time GPs, administrative staff and no practice nurse. - Much of the practice management was handled by the owner GP and there was a high degree of turnover of administrative staff. - Mean TCI score was above average when compared to other cases especially in ‘participant safety’ and ‘vision’; however, job satisfaction was similar to other cases. - The owner GP had a strong interest in use of software interventions to improve health care quality, however, there was little formal participation in QI programs prior to participation in this research project. - Total support time was 12 hours (mostly IT support) and this was mainly during the trial period with minimal support provided in the post-trial period. - Trial outcomes were significantly improved over the trial period for both screening and prescribing and then plateaued and slightly declined in the post-trial period for screening. |
| --- |

**Case 3.**

| - A solo GP practice with a full-time manager/receptionist in a diverse Sydney community near a major business district. - The GP had been in practice for over twenty years but had little experience with QI programs. - The mean TCI and job satisfaction scores for the two staff members were higher than average when compared to all cases and sites. - Initial implementation of intervention and training to this GP was provided by the GP principal investigator and only one additional onsite training visit was provided by the research team. - Total support time was approximately 3 hours. - Trial outcomes were above average at baseline. Screening rates decreased but overall remained broadly unchanged by the end of the post-trial period. There were improvements in prescribing rates over both the trial and post-trial periods. |
| --- |

**Case 4.**

| - A mid-sized teaching general practice in a rural outer Western Sydney suburb. - The owner GP established the practice over 30 years ago and has been active in supervising GP registrar training for many years. There is a full-time practice manager, part-time nurse and up to three full-time training GP registrars who generally did 6-month placements at the practice. - The mean TCI and job satisfaction scores were higher than the average score for the cases and other sites. - Despite extensive involvement in teaching and supervision, the practice has not been involved in any formal QI programs. - There was extensive onsite training provided by project officers throughout the trial period with frequent refresher courses when new GP registrars commenced at the practice. Total support time was approximately 11 hours. - Trial outcomes were below average at baseline. Screening rates improved substantially over the trial period but deteriorated in the post-trial period. There were steady improvements in prescribing rates over both the trial and post-trial periods. |
| --- |

**Case 5.**

| - A large ACCHS in a remote region with strong Aboriginal community representation on its all Aboriginal governing board. - The service was over 20 years old and received funding from both state and federal governments. - It had a chronic disease strategy in place for 12 years which included a strong focus on CQI programs. - Initial training was conducted by the GP principal investigator over three separate sessions for a total of 23 staff including GPs, nurses, Aboriginal Health Workers (AHWs) and a health information officer (HIO). Additional support was provided via webinar and phone when required over the course of the trial and minimal support in the post-trial period. Total support time was approximately 12.5 hours. - TCI and job satisfaction scores were lower than average compared to other cases, especially in the TCI subgroup regarding ‘Vision’ and ‘Task Orientation’. The health service had a high baseline performance in the trial outcomes reflective of its extensive involvement in CQI programs. - Trial outcomes in prescribing increased during the trial period and subsequently plateaued, and screening stayed relatively unchanged. |
| --- |

**Case 6.**

| - A large urban ACCHS in the state of Queensland. The service has two clinic locations with the main branch in a mixed residential and commercial area; and a smaller clinic located in a shopping centre. - The service was over 20 years old and is governed by an all Aboriginal and Torres Strait Islander board of directors. - There were around 26 staff in total with 5 GPs (3 full-time and 2 part-time). - The service had been regularly involved in a CQI collaborative run by an external state-based organisation representing Aboriginal and Torres Strait Islander health services but at the time of the study, this program was not actively being implemented. - Initial implementation of intervention and training was conducted by two project officers in a group forum and then individually to each staff member. Additional training support was provided via webinar and phone when required over the course of the trial. - As with Case 5, TCI and job satisfaction scores were lower than average compared to other cases, especially in the TCI subgroup regarding ‘Vision’ and ‘Task Orientation’. - Total support time was 17 hours. - Slightly above average performance in screening and prescribing was observed at baseline and this largely remained unchanged throughout the trial and post-trial periods. |
| --- |
