# Supplementary material for: What drives adoption of a computerised, multifaceted quality improvement intervention for cardiovascular disease management in primary healthcare settings? A mixed methods analysis using normalisation process theory
Source: Implement Sci. 2018 Nov 12;13:140. doi: 10.1186/s13012-018-0830-x (PMC6233504; doi:10.1186/s13012-018-0830-x)
Supplement: Supplementary file 5 — Health professional interview findings and quotes. (DOCX 83 kb) [file 13012_2018_830_MOESM5_ESM.docx]

| TORPEDOHealth Professionals Interview Guide  \| **DATE** \| **Health Service Name** \| **Health Service #** \| **Participant Code** \| **Interviewer Name** \| \| --- \| --- \| --- \| --- \| --- \| \| / / 13 \|  \|  \|  \|  \|   **Job Title……………………………………………………………………..............**  **Locations currently working at:…………………………………………..............**  **Do you have access to HealthTracker-CVD tool on your computer?** Yes/No (if yes, answer question below) |
| --- | --- | --- | --- | --- | --- | --- | --- | --- | --- | --- |
| **Sampling Matrix:**   \| **Type of Health Service** \| General Practice (GPs) \| \| \| \| \| \| Aboriginal Community Controlled Health Service (ACCHSs) \| \| \| \| \| Urban \| \| \| \| \| \| --- \| --- \| --- \| --- \| --- \| --- \| --- \| --- \| --- \| --- \| --- \| --- \| --- \| --- \| --- \| --- \| --- \| \| Rural \| \| \| \| \| \| **EHR** \| Medical Director 🞏 \| \| \| \| \| \| Best Practice 🞏 \| \| \| \| \| \| \| \| \| \| \| **Change in CVD screening Outcome** \| Large (>10% ) \| \| Medium (5-10%) \| \| Small to None (<5%) \| \| Large (>10%) \| \| \| Medium (5-10%) \| \| \| \| Small to none (<5%) \| \| \| \| **Complexity of HS**  **staff** \| # of GPs \| # Full time \| PM (Y/N) \| # of PNs \| \| # Full time \| # of GPs \| # Full time \| PM/(Manager type) (Y/N) \| \| # of Nurses \| \| # Full time \| \| # of Healthworkers \| # Full time \| \| # Part-time \| # Part time \| # Part time \| # Part time \| \| # Part time \|   **Social-demographic**   \| Age \| **20-29** \| **30-39** \| **40-49** \| **50-59** \| **60-69** \| **70+** \| \| --- \| --- \| --- \| --- \| --- \| --- \| --- \| \| Gender \|  \| \| \| \| \| \| \| First language \|  \| \| \| \| \| \| \| Other languages \|  \| \| \| \| \| \| \| Country of medical graduation.  Postgraduate medical qualifications \|  \| \| \| \| \| \| \| Years worked in general practice \|  \| \| \| \| \| \| \| Years worked in Aboriginal Health \|  \| \| \| \| \| \| \| Length of time at current ACCHS/General practice \|  \| \| \| \| \| \|   **Social-demographic AHW, Nurse, and other Health Profesionals**   \| Age \| **20-29** \| **30-39** \| **40-49** \| **50-59** \| **60-69** \| **70+** \| \| --- \| --- \| --- \| --- \| --- \| --- \| --- \| \| Gender \|  \| \| \| \| \| \| \| First language \|  \| \| \| \| \| \| \| Other languages \|  \| \| \| \| \| \| \| Time in practicing as Nurse, AHW, other HP \|  \| \| \| \| \| \| \| Time at site \|  \| \| \| \| \| \| \| Highest level of education \| Year 10 or below  Year 11-12  College diploma or similar  Undergraduate university degree  Postgraduate university degree \| \| \| \| \| \| \| Formal Cross cultural training \| CALD  Indigenous \| \| \| \| \| \|   **Notes** |

|  |
| --- |

# TORPEDO - Health Professionals Interview Guide

| **Area of Interest** | **Initial Broad Descriptive Questions** | **Possible Probing Questions (These are a guide only. It is not expected that you ask all these questions)** |
| --- | --- | --- |
| **[1]**  **Views of the reason for outcomes** | **Reviewing your results from your health service, what do you think might be the reason for these outcomes?**  **Now, let’s take a look at your data in CAT. We will then open up your last data extraction, and review your data?** | - show them their randomization results and their monthly progress from the IF portal data (print out EOS feedback report) - Did you use the TORPEDO portal for viewing your data? Why/why not? - How often did you use the IF portal? - If you did use it, how useful did you find it? - What benefits did the IF portal have at your practice or health service? - Do you see a value in using IF portal? What would influence your use of the portal more often? - How did you use the TORPEDO portal feedback reports given to you by the project officers? How was this discussed with the team or other GPs involved? - Did you use the CAT? Why/why not? - If you did use it, how useful was it? How often did you use it? - Did you use it only for HT or did you use it for other health outcomes and data quality information? - For what purpose would you use CAT at your health service? Who would be the main person using CAT? How would information be relayed to the team at the Health Service? |
| **[2a]**  **Use of HealthTracker-CVD tool**  **[2b] Use of Risk Projection graph**  **[2c] Implementation of Guidelines** | **Can you remember the last patient you used HealthTracker for? And how did you use HealthTracker for this patient?**  **How did you use the risk communication graph for your last patient?**  **To what extent were you using the National Vascular Disease Prevention Alliance Guidelines in patient care before participating in our Study?** | - What purpose do you use HT for? - Roughly how often do you use HT in a day for your patients? - What kinds of patients do you use HT with? - How was using the HealthTracker tool at point of care different from when you would not use the tool? Did you at a later time assess if use of HealthTracker improved care?How long did it take you to use the tool confidently? How confident are you in your knowledge and skills in using HealthTracker-CVD? - What do you see as major advantages of using the HT tool? - What do you see as the major disadvantages or barriers of using the HT tool? - Did you use the risk projection graphs? Why/why not? - If you did use it, what did the patient think of the “What if” graphs? - How did the patients find the print outs of their risk summary and recommendations? - Did you give your patients print outs of resources? What type of impact do you think the risk summary and resources print out has on patients (if applicable)? - At a later visit from the patient, did you review again with the patients their risk score? - What recommendations in the guidelines were you implementing and using? - Are you aware of the new updated NVPDA guidelines on absolute risk management? If yes, what are your thoughts on the guidelines? - How did you generally access the NVDPA guideline? Hardcopy, electronically, other doctors, meeting, etc? - How has HealthTracker helped you to use NVDPA guideline? Or any other guidelines used in HealthTracker? - What knowledge in relation to the NVPDA guideline or any other guidelines have you gained after using HealthTracker? |
| **[3]-** *For GPs Only*  **Knowledge and use of absolute risk calculation and management** | **What are your views about absolute risk calculation in the management of your patients?**  **How have they changed in your prescribing methods as a consequence of taking part in TORPEDO?** | - Have you been using absolute risk calculations to calculate patients estimated risk of heart attack or stroke? If yes, how do you find this useful in managing/treating patients CVD? - How did absolute risk score in HealthTracker-CVD facilitate treatment or care of the patient? How would you normally treat patients if you did not have access to HealthTracker? - What are your thoughts on prescribing to high risk patients who do not have established disease? - What are your thoughts on prescribing to low risk patients who may have high BP or cholesterol who do not have established disease? - How do you prescribe to those patients you have co-morbidities? - How do you handle patients who need blood pressure and/or cholesterol medication and do not want to take any medication? What steps do you take to help them understand their condition when they don’t have an visible systoms? |
| **[5a]**  **Support/training**  **[5b]**  **Prior knowledge and skills of electronic health technology** | **What barriers did you/your practice face in taking part in the study? In using HT?**  **What were the facilitators?**  **Had you used any e-health tools, such as SideBar and Clinical Audit, Doctor’s Portal, before taking part in TORPEDO?**  **Have you started using any e-health tools since then?** | Software:   - Were there any issues installing SideBar on your practice staffs’ computers - Once SideBar was installed, did you have any issues using HealthTracker-CVD? - How did SideBar perform after any updates from PEN Computer systems? How did the update affect you? - What types of issues, if any, did you have overall with performance of SideBar and/or HealthTracker tool?   Support Training:   - Were you provided with enough information and support from study staff to confidently use the tool? If not, what do you think you would have wanted to happen? - How did the initial training impact your use of the tool? - How did you find the support/training from the study team? Who at the health service was dedicated to work with the study team, resolving issues, and communicating to software provider if needed?   Prior use of e-health tools:   - If yes, which tools and how long have you been using them? - How long have you been using electronic health records? - Do you have knowledge of electronic decision support tools? If yes, what have you heard about EDS tools? - How confident are you in general with new computer programs? |
| **[6] Workability and integration of HealthTracker-CVD at practice/HS** | **What helped you/your practice take part in the study? How was HealthTracker-CVD integrated within your practice/HS?** | Staff at Health Service:   - How do you work with other GPs and staff at your Health Service? (if applicable) - What incentives do GPs receive to participate in studies? - What type of support is given to non-GP staff? Are there incentives at your health service for the staff? If yes, what types of incentives?      - What was the % of staff turnover in the last year? - At your health service, do you have regular meetings with team? If yes, what is discussed at these meetings?   Resources:   - Did you have competing work demands and time constraints while participating in the study? If yes, how did you handle competing work demands and time constraints? - What type of financial support do you receive at your health service, if any? - Do you have any external management groups involved at your health service? - How would you describe your financial stability at your health service?   Involvement of programs to improve health services:   - What types of continuous quality improvement programs is your health service involved in? - What are your thoughts on continuous quality improvement programs? - If you have not been involved in continuous quality improvement programs, would you be interested in participating? How do you think this will impact your health service? What types of incentives would encourage you to participate in CQI programs? |
| **[7]**  **General Impact of the Study on the health service?**  *To understand how the study integrated into everyday practice* | **In what ways do you think the ACCHS/general practice (in which you work) has changed as a result of participating in the study?** | - What was the impact on you and your health service in choosing to be a part of this study? - Were there any benefits to you and your health service of participating in the study? If so please explain? - Were there any problems with you and your health service in participating in the study? If so please explain? - What impact did HealthTracker have on the consultation process? - How did it impact on the length of the consultation? |
| **[8]-***For GPs Only*  **Motivation to participate** | **What were your reasons for taking part in the TORPEDO study?** | - Were there any benefits to you and your practice in participating in the study? If so, explain? - Do you think you would take part in a similar study in future? - What factors would make you more likely to take part? - What factors would make you less likely to take part? |
| **[9]-***For GPs Only*  **Attitudes to e-health** | **E-health is becoming a major government priority.** | - What are your thoughts on electronic decision support tools as best practice use in primary health care? - What types of resources, support, and training do you think would be needed to change primary health care to use more e-health tools? - What advice would you give to government on implementation of HealthTracker-CVD in primary health care? |
| **[9]**  **Final comments** | - Is there anything else you would like to add that we have not discussed in this interview? | |

Note: Some questions in the guidelines will be modified in response to participant’s answers and themes emerging from the data.

**Thank you for your participation.**

**Notes:**
